# An Efficient GPU-Accelerated High-Order Upwind Rotated Lattice Boltzmann Flux Solver for Simulating Three-Dimensional Compressible Flows with Strong Shock Waves

**DOI:** 10.3390/e27121193

**Published:** 2025-11-24

**Authors:** Yunhao Wang, Qite Wang, Yan Wang

**Affiliations:** 1 College of Aerospace Engineering, Nanjing University of Aeronautics and Astronautics, Yudao Street 29, Nanjing 210016, China; 2 College of Civil Aviation, Nanjing University of Aeronautics and Astronautics, Yudao Street 29, Nanjing 210016, China; 3 China Aerospace Times Feihong Technology Co., Ltd., China Academy of Aerospace Electronics Technology, Intelligent Unmanned System Overall Technology Research and Development Center, Beijing 100094, China

**Keywords:** WENO-URLBFS, GPU parallel, 3D compressible flow, high order, low numerical dissipation

## Abstract

This paper presents an efficient and high-order WENO-based Upwind Rotated Lattice Boltzmann Flux Solver (WENO-URLBFS) on graphics processing units (GPUs) for simulating three-dimensional (3D) compressible flow problems. The proposed approach extends the baseline Rotated Lattice Boltzmann Flux Solver (RLBFS) by redefining the interface tangential velocity based on the theoretical solution of the Euler equations. This improvement, combined with a weighted decomposition of the numerical fluxes in two mutually perpendicular directions, effectively reduces numerical dissipation and enhances solution stability. To achieve high-order accuracy, the WENO interpolation is applied in the characteristic space to reconstruct physical quantities on both sides of the interface. The density perturbation test is employed to assess the accuracy of the scheme, which demonstrates 5th- and 7th-order convergence as expected. In addition, this test case is also employed to confirm the consistency between the CPU serial and GPU parallel implementations of the WENO-URLBFS scheme and to assess the acceleration performance across different grid resolutions, yielding a maximum speedup factor of 1208.27. The low-dissipation property of the scheme is further assessed through the inviscid Taylor–Green vortex problem. Finally, a series of challenging three-dimensional benchmark cases demonstrate that the present scheme achieves high accuracy, low dissipation, and excellent computational efficiency in simulating strongly compressible flows with complex features such as strong shock waves and discontinuities.

## 1. Introduction

Advances in numerical methods and computing resources have significantly propelled the advancement of computational fluid dynamics (CFD), making it widely applicable in various fields [1]. Compressible flow problems in aerospace often feature complex phenomena like strong shocks and contact discontinuities, requiring numerical methods with greater accuracy and efficiency. However, traditional low-order numerical methods usually fail to accurately capture these complex features, resulting in insufficient simulation results. In addition, with the increase in problem dimensions and computational scale, the consumption of computing resources also increases sharply, and a single serial program makes it difficult to meet practical needs. Therefore, developing a high-precision, low-dissipation numerical method with efficient computational capabilities is particularly important.

The key to constructing a high-precision, low-dissipation numerical scheme lies in the effective application of interpolation reconstruction techniques and the rational selection of flux evaluation methods. The traditional second-order interpolation method, such as the Monotonic Upstream-Centered Schemes for Conservation Laws (MUSCL) difference scheme [2] and the total variation reduction (TVD) scheme [3,4,5,6], can simulate contact discontinuity problems to a certain extent. However, these schemes often introduce significant numerical dissipation when dealing with strong discontinuities or complex flow field structures, resulting in excessive smoothing of detailed flow field structures. Although these numerical schemes effectively prevent non-physical oscillations near contact discontinuities, their ability to capture details in high-gradient regions is relatively limited, thus restricting the overall accuracy and resolution of the computation. To address these challenges, researchers have developed advanced numerical schemes to effectively capture complex flow characteristics. Harten [7] introduced the essentially non-oscillatory (ENO) scheme, which theoretically achieves arbitrary high-order accuracy. Shu and Osher [8,9] introduced a highly accurate ENO scheme within the FDM framework. The ENO scheme’s reliance on selecting multiple stencils for each calculation leads to a loss of physical information and reduced computational efficiency. Liu et al. [10] developed the weighted essentially non-oscillatory (WENO) scheme to solve this problem by adopting a nonlinear combination of reconstruction functions for all candidate templates, thereby constructing a finite-volume WENO scheme of 3rd-order accuracy. Jiang and Shu [11] refined the smoothness indicators and established the foundational framework for finite difference WENO (FD-WENO) schemes in multidimensional spaces, culminating in the classic WENO-JS scheme, which achieves fifth-order numerical accuracy. Building on this, many WENO schemes have been proposed [12,13]. The finite difference method has become preferred for constructing high-precision numerical schemes due to its simple and efficient parallel capabilities and the convenience of high-order accuracy expansion.

After adopting the appropriate interpolation reconstruction technique, the choice of flux evaluation method is also crucial. The evaluation of flux is crucial to guarantee the stability and accuracy of numerical calculations, particularly when handling shock waves, discontinuities, and intricate flow phenomena. Therefore, a reasonable flux evaluation method can effectively control numerical dissipation and enhance the precision of the solution. The following will introduce several commonly used flux evaluation methods and their performance in practical applications. Typical numerical schemes, such as the Lax-Friedrichs (LF) [14], ROE [15], and the HLL scheme [16], are primarily used for directly approximating the macroscopic flux. However, the LF scheme exhibits significant numerical dissipation, affecting the accuracy of the results. When simulating high-speed flow problems with strong shock waves, the ROE and HLL schemes can exhibit various forms of instability, particularly carbuncle phenomena [17,18,19,20]. A mesoscopic lattice Boltzmann method (LBM) [21,22,23] has gradually become prevalent in recent years, mainly for incompressible flows. The solver employs the local reconstruction of the LBM to evaluate numerical fluxes at the interface. It performs well under low-speed inflow conditions but has limitations when applied to high-speed flows. People have changed the equilibrium distribution function to apply to compressible flows [24,25,26,27,28], but the new equilibrium distribution function is relatively complex, which affects the computational efficiency. To this end, Shu et al. [29,30,31] developed the lattice Boltzmann flux solver (LBFS), and Yang et al. [32,33] advanced the method by proposing an optimized D1Q4 model to improve computational efficiency. Chen [34] proposed a RLBFS, which decomposes the interface normal vector into two orthogonal components, calculates the directional flux separately, and generates a new numerical flux by weighted combination. This method has good numerical stability, but the accuracy is only second order. It should be emphasized that in both LBFS and RLBFS, the tangential velocity is calculated approximately, which can lead to discrepancies with the theoretical solution of the Euler equations [35]. Such approximations often introduce additional numerical dissipation, resulting in a reduction in the resolution of the results. This is the primary motivation for the investigation of low-dissipation numerical schemes in this paper.

With the increase in problem dimensionality, the computation time increases exponentially. Traditional serial computation methods cannot meet the needs of real-time computation; so, parallel acceleration computation is needed. Initially, computational acceleration relied on multiple processor central processing units (CPU) to achieve parallel computing [36,37]. Although efficiency can be improved to a certain extent, as the scale of calculation increases, CPU performance is limited by the number of cores and processing power, and performance bottlenecks will gradually emerge, especially when processing large-scale data and floating-point operations. With the rapid development of GPU technology, GPU-based parallel computing methods have gradually become more efficient acceleration approaches. GPU has significant advantages in processing large-scale parallel computing tasks compared to the CPU. Its vast number of computing cores and powerful parallel processing capabilities enable the GPU to quickly handle many computing tasks simultaneously, significantly improving the computing speed. GPU acceleration has shown unparalleled advantages in CFD calculations with high resolution and complex physical phenomena [38,39,40,41,42,43]. In order to make full use of computing resources, a heterogeneous computing paradigm is adopted to divide the computing tasks into parts suitable for GPU and CPU [44,45,46]. Under this architecture, the GPU is responsible for computing-intensive tasks such as numerical flux calculation and time advancement, while the CPU is mainly responsible for data reading and relatively light tasks. Specifically, a single NVIDIA TITAN V executes the GPU procedure, and the CPU procedure is executed on the Hygon 7185.

In this paper, the URLBFS as a new numerical scheme, is introduced by improving the calculation method of the interface tangential velocity. Combine the FDM framework with the WENO higher-order method, the 5th order and 7th order FD-WENO-URLBFS schemes are constructed, which can accurately simulate the complex flow characteristics in compressible flows and effectively reduce numerical dissipation. The scheme is accelerated by GPU using the CUDA platform, which greatly improves the computational efficiency. Section 2 of this article is the methodology, Section 3 discusses GPU implementations, Section 4 assesses the precision and computational speed of the numerical method through challenging 3D test cases and Section 5 is the conclusion.

## 2. Methodology

### 2.1. Governing Equations

The conservation form of the 3D Navier–Stokes (NS) equation is shown below:

(1) $$\frac{\partial W}{\partial t}+\frac{\partial F_{c}}{\partial x}+\frac{\partial G_{c}}{\partial y}+\frac{\partial H_{c}}{\partial z}=\frac{\partial F_{v}}{\partial x}+\frac{\partial G_{v}}{\partial y}+\frac{\partial H_{v}}{\partial z},$$

(2) $$\begin{array}{c} W=\left[\begin{array}{c} \rho \\ \rho u\\ \begin{array}{l} \rho v\\ \rho e \end{array}\\ E \end{array}\right],\;F_{c}=\left[\begin{array}{c} \rho u\\ \rho u^{2}+p\\ \begin{array}{l} \rho uv\\ \rho uw \end{array}\\ u(E+p) \end{array}\right],\;G_{c}=\left[\begin{array}{c} \rho v\\ \rho vu\\ \begin{array}{l} \rho v^{2}+p\\ \rho vw \end{array}\\ v(E+p) \end{array}\right],\;H_{c}=\left[\begin{array}{c} \rho w\\ \rho wu\\ \begin{array}{c} \rho wv\\ \;\rho w^{2}+p \end{array}\\ w(E+p) \end{array}\right],\\ F_{v}=\left[\begin{array}{c} 0\\ \tau_{xx}\\ \tau_{xy}\\ \tau_{xz}\\ u\tau_{xx}+v\tau_{xy}+w\tau_{xz}+\kappa \frac{\partial T}{\partial x} \end{array}\right],G_{v}=\left[\begin{array}{c} 0\\ \tau_{yx}\\ \tau_{yy}\\ \tau_{yz}\\ u\tau_{yx}+v\tau_{yy}+w\tau_{yz}+\kappa \frac{\partial T}{\partial y} \end{array}\right],H_{v}=\left[\begin{array}{c} 0\\ \tau_{zx}\\ \tau_{zy}\\ \tau_{zz}\\ u\tau_{zx}+v\tau_{zy}+w\tau_{zz}+\kappa \frac{\partial T}{\partial z} \end{array}\right]. \end{array}$$

where ρ represents the density, p and T correspond to pressure and temperature, u, v, w denote the velocity components along each of the three axes. The viscosity tensor τ is:

(3) $$\tau =\mu \left[\begin{array}{ccc} \frac{4}{3}\frac{\partial u}{\partial x}-\frac{2}{3}\left(\frac{\partial v}{\partial y}+\frac{\partial w}{\partial z}\right) & \frac{\partial u}{\partial y}+\frac{\partial v}{\partial x} & \frac{\partial u}{\partial z}+\frac{\partial w}{\partial x}\\ \frac{\partial v}{\partial x}+\frac{\partial u}{\partial y} & \frac{4}{3}\frac{\partial v}{\partial y}-\frac{2}{3}\left(\frac{\partial u}{\partial x}+\frac{\partial w}{\partial z}\right) & \frac{\partial v}{\partial z}+\frac{\partial w}{\partial y}\\ \frac{\partial w}{\partial x}+\frac{\partial u}{\partial z} & \frac{\partial w}{\partial y}+\frac{\partial v}{\partial z} & \frac{4}{3}\frac{\partial w}{\partial z}-\frac{2}{3}\left(\frac{\partial u}{\partial x}+\frac{\partial v}{\partial y}\right) \end{array}\right].$$

μ represents the molecular viscosity. $\kappa =\mu C_{p}/\mathrm{Pr}$, which represents the thermal conductivity, where $C_{p}$ means the specific heat at constant pressure, and Pr is the Prandtl number. E represents the total energy, as shown below:

(4) $$E=\rho \left(\frac{1}{2}V^{2}+e\right),$$

where e represents internal energy. The kinetic energy of the fluid is:

(5) $$\frac{1}{2}V^{2}=\frac{1}{2}\left(u^{2}+v^{2}+w^{2}\right),$$

The introduction of the state equation completes the problem described by the Euler equation, as shown below:

(6) $$p=\left(\gamma -1\right)\left[E-\frac{1}{2}\rho \left(u^{2}+v^{2}+w^{2}\right)\right].$$

Here, γ is the specific heat ratio of air. Unless otherwise specified, all examples in this paper take the value of γ=1.4.

All examples in this paper are spatially discretized in a uniform grid, where Δx=Δy=Δz. The FDM method is used to discretize Equation (1), and the semi-discrete equation at the discrete point is:

(7) $$\frac{dW_{ijk}(t)}{dt}=-\frac{1}{\Delta x}\left(F_{i+1/2,j,k}-F_{i-1/2,j,k}\right)-\frac{1}{\Delta y}\left(G_{i,j+1/2,k}-G_{i,j-1/2,k}\right)-\frac{1}{\Delta z}\left(H_{i,j,k+1/2}-H_{i,j,k-1/2}\right).$$

where $W_{ijk}(t)$ is a numerical approximation of W(i,j,k,t). $F=F_{c}-F_{v}$, $G=G_{c}-G_{v}$ and $H=H_{c}-H_{v}$ are numerical fluxes in three directions, respectively. The ordinary differential Equation about time is written as follows:

(8) $$\frac{dW(t)}{dt}=L(h),$$

The third-order TVD Runge–Kutta method is commonly employed due to its strong stability properties. It is formulated as follows:

(9) $$\begin{array}{c} h^{\left(1\right)}=h^{n}+\Delta tL(h^{n}),\\ h^{\left(2\right)}=\frac{3}{4}h^{n}+\frac{1}{4}h^{\left(1\right)}+\frac{1}{4}\Delta tL(h^{\left(1\right)}),\\ h^{n+1}=\frac{1}{3}h^{n}+\frac{2}{3}h^{\left(2\right)}+\frac{2}{3}\Delta tL(h^{\left(2\right)}). \end{array}$$

This scheme preserves TVD properties and can suppress numerical oscillations, making it suitable for solving complex flow phenomena in high mach flow problems. The velocity and temperature gradients in the governing equations via second-order central difference scheme to compute the stress tensor and heat flux, from which the viscous numerical flux is then determined. In the following section, the focus will be on the evaluation methods for the inviscid numerical fluxes.

### 2.2. Inviscid Numerical Flux Evaluation Method

This section begins by reviewing the traditional RLBFS and then introduces an enhanced version, the URLBFS. The primary improvement in the URLBFS is the redefinition of the tangential velocity at the interface. Instead of relying on approximate methods, this improvement uses the exact Euler-equation solution to calculate the tangential velocity, which helps to reduce numerical dissipation significantly.

#### 2.2.1. The RLBFS Scheme

**Figure 1:**
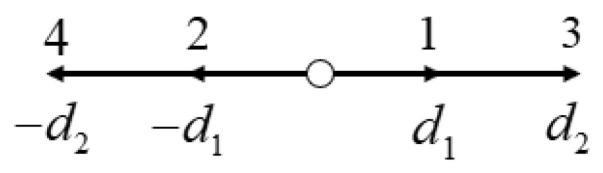
Distribution of the D1Q4 lattice velocity model.

**Figure 2:**
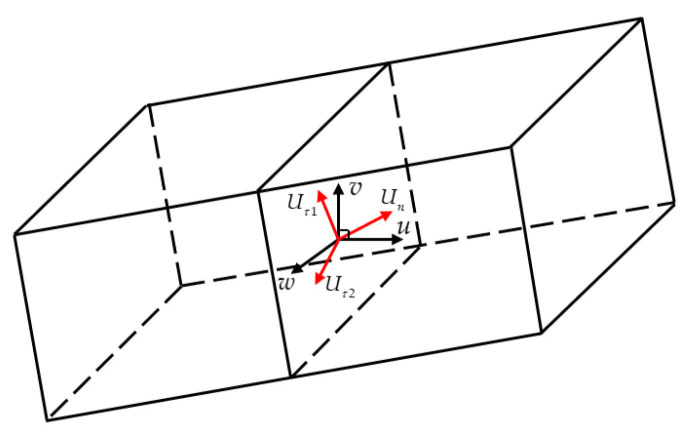
Use of the 1D LB Model at Cell Interfaces in 3D Simulations. Black arrows represent the velocity components in the global Cartesian frame, and red arrows denote the velocity components in the local rotated coordinate system.

Chen [47] proposed the RLBFS method, which decomposes the interface normal vector into two perpendicular components. Subsequently, directional numerical fluxes are obtained using the LBFS scheme with a D1Q4 lattice Boltzmann (LB) model, and these fluxes are merged via a weighted procedure to form the final numerical flux. The D1Q4 model overcomes the problem of performance degradation of LBFS schemes in other one-dimensional models due to too many user-specified parameters [24,32]. The model is shown in Figure 1. The equilibrium distribution function $g_{m}\left(m=1,2,3,4\right)$ and lattice velocity $d_{m}\left(m=1,2\right)$ can be expressed as follows:

(10) $$\begin{array}{c} g_{1}=\frac{\rho (-d_{1}d_{2}^{2}-d_{2}^{2}u+d_{1}u^{2}+d_{1}\vartheta^{2}+u^{3}+3u\vartheta^{2})}{2d_{1}(d_{1}^{2}-d_{2}^{2})},\\ g_{2}=\frac{\rho (-d_{1}d_{2}^{2}+d_{2}^{2}u+d_{1}u^{2}+d_{1}\vartheta^{2}-u^{3}-3u\vartheta^{2})}{2d_{1}(d_{1}^{2}-d_{2}^{2})},\\ g_{3}=\frac{\rho (d_{1}^{2}d_{2}+d_{1}^{2}u-d_{2}u^{2}-d_{2}\vartheta^{2}-u^{3}-3u\vartheta^{2})}{2d_{2}(d_{1}^{2}-d_{2}^{2})},\\ \begin{array}{l} g_{4}=\frac{\rho (d_{1}^{2}d_{2}-d_{1}^{2}u-d_{2}u^{2}-d_{2}\vartheta^{2}+u^{3}+3u\vartheta^{2})}{2d_{2}(d_{1}^{2}-d_{2}^{2})},\\ d_{1}=\sqrt{u^{2}+3\vartheta^{2}-\sqrt{4u^{2}\vartheta^{2}+6\vartheta^{4}}},\\ \;d_{2}=\sqrt{u^{2}+3\vartheta^{2}+\sqrt{4u^{2}\vartheta^{2}+6\vartheta^{4}}}, \end{array} \end{array}$$

where $\vartheta =\sqrt{Dp/\rho }$ is the peculiar velocity, and D represents the spatial dimension, which is 1 in this paper. When $g_{m}$ and $d_{m}$ are determined, the variables in Equation (1) are given by:

(11) $$\begin{array}{c} \rho =\sum_{i}g_{i},\\ \rho u=\sum_{i}g_{i}\xi_{i},\\ \rho uu+p=\sum_{i}g_{i}\xi_{i}\xi_{i},\\ \rho E=\sum_{i}g_{i}(\frac{1}{2}\xi_{i}\xi_{i}+e_{p}),\\ (\rho E+p)u=\sum_{i}g_{i}\left(\frac{1}{2}\xi_{i}\xi_{i}+e_{p}\right)\xi_{i}, \end{array}$$

where $\xi_{i}$ represents the particle velocity, $\xi_{1}=d_{1},$$\xi_{2}=-d_{1},$$\xi_{3}=d_{2},$$\xi_{4}=-d_{2}$, $e_{p}=[1-\frac{D}{2}(\gamma -1)]e$ and $e=p/\left[\left(\gamma -1\right)\rho \right]$. When applying the above D1Q4 model to solve problems involving multiple dimensions, it is usually only considered to apply this method along the interface normal. However, for a three-dimensional case, as depicted in Figure 2, this paper replaces the velocity u in Equation (10) with the normal velocity $U_{n}=U\cdot n$. The tangential velocity vector is $U_{\tau }=\left(u_{\tau x},u_{\tau y},u_{\tau z}\right)=u-U_{n}n$. Then, the variables W and convective flux $F_{c}$ in Equation (2) are:

(12) $$\begin{array}{l} \left.W=\left[\begin{array}{c} \rho \\ \rho \left(U_{n}n_{x}+u_{\tau x}\right)\\ \begin{array}{l} \rho \left(U_{n}n_{y}+u_{\tau y}\right)\\ \rho \left(U_{n}n_{z}+u_{\tau z}\right) \end{array}\\ \rho \left(U_{n}^{2}/2+e\right)+\rho U_{\tau }^{2}/2 \end{array}\right.\right],\\ \left.F_{c}=\left[\begin{array}{c} \rho U_{n}\\ \left(\rho U_{n}^{2}+p\right)n_{x}+\rho U_{n}u_{\tau x}\\ \begin{array}{l} \left(\rho U_{n}^{2}+p\right)n_{y}+\rho U_{n}u_{\tau y}\\ \left(\rho U_{n}^{2}+p\right)n_{z}+\rho U_{n}u_{\tau z} \end{array}\\ \left(\rho \left(U_{n}^{2}/2+e\right)+p\right)U_{n}+\rho U_{n}U_{\tau }^{2}/2 \end{array}\right.\right]. \end{array}$$

Considering only the normal velocity, the inviscid flux at the cell interface x=0 is:

(13) $$\begin{array}{cc} {\left(F_{c}\right)}_{i+1/2}^{*}={\left[\begin{array}{ccc} \rho U_{n} & \rho U_{n}U_{n}+p & \left(\rho \left(\frac{1}{2}U_{n}U_{n}+e\right)+p\right)U_{n} \end{array}\right]}^{T} & =\sum_{i}\xi_{i}\varphi_{\alpha }f_{i}(0,t), \end{array}$$

where $\varphi_{\alpha }={\left(1,\xi_{i},\frac{1}{2}\xi_{i}^{2}+e_{p}\right)}^{T}$ and the superscript * indicates numerical value. $f_{i}(0,t)$ is given by:

(14) $$f_{i}(0,t)=g_{i}(0,t)-\tau_{0}[g_{i}(0,t)-g_{i}(-\xi_{i}\delta t,t-\delta t)]+O(\delta t),$$

$g_{i}(0,t)$ and $g_{i}(-\xi_{i}\delta t,t-\delta t)$ represent the equilibrium distribution functions at the interface and at adjacent points. The numerical flux accounting only for the normal direction is:

(15) $$\begin{array}{ll} {\left(F_{c}\right)}_{i+1/2}^{*} & =\left(1-\tau_{0}\right)\sum_{i}\xi_{i}\varphi_{\alpha }g_{i}\left(0,t\right)+\tau_{0}\sum_{i}\xi_{i}\varphi_{\alpha }g_{i}\left(-\xi_{i}\delta t,t-\delta t\right)\\ & =\left(1-\tau_{0}\right)F_{c,i+1/2}^{\left(I*\right)}+\tau_{0}F_{c,i+1/2}^{\left(II*\right)}. \end{array}$$

where $\tau_{0}=\tau /\delta t$ is the dimensionless collision time. Inserting Equation (14) into Equation (13) and including the effect of tangential velocity, the complete numerical flux takes the form:

(16) $$\left.F_{c,i+1/2}^{\left(I\right)}=\left(1-\tau_{0}\right)\left[\begin{array}{c} F_{c,i+1/2}^{\left(I*\right)}(1)\\ F_{c,i+1/2}^{\left(I*\right)}(2)n_{x}+\rho U_{n}u_{\tau x}\\ F_{c,i+1/2}^{\left(I*\right)}(2)n_{y}+\rho U_{n}u_{\tau y}\\ F_{c,i+1/2}^{\left(I*\right)}(2)n_{z}+\rho U_{n}u_{\tau z}\\ F_{c,i+1/2}^{\left(I*\right)}(3)+\rho U_{n}U_{\tau }^{2}/2 \end{array}\right.\right]\;+\tau_{0}\left.\left[\begin{array}{c} F_{c,i+1/2}^{\left(ll*\right)}(1)\\ F_{c,i+1/2}^{\left(ll*\right)}(2)n_{x}+\rho U_{n}u_{\tau x}\\ F_{c,i+1/2}^{\left(ll*\right)}(2)n_{y}+\rho U_{n}u_{\tau y}\\ F_{c,i+1/2}^{\left(ll*\right)}(2)n_{z}+\rho U_{n}u_{\tau z}\\ F_{c,i+1/2}^{\left(ll*\right)}(3)+\rho U_{n}U_{\tau }^{2}/2 \end{array}\right.\right].$$

**Figure 3:**
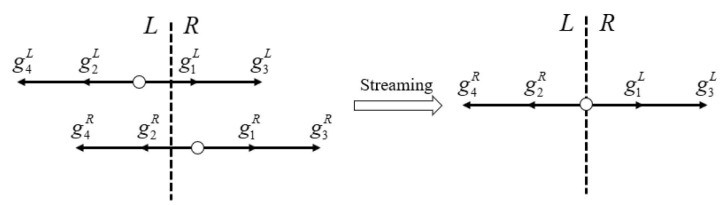
Streaming process of D1Q4 model.

From the above equation, it is clear that the key step in obtaining the inviscid numerical flux is the $g_{i}(0,t)$ and $g_{i}(-\xi_{i}\delta t,t-\delta t)$. Function $g_{i}(-\xi_{i}\delta t,t-\delta t)$ can be solved by:

(17) $$g_{i}(-\xi_{i}\delta t,t-\delta t)=\left\{\begin{array}{c} g_{i}^{L},\;\mathrm{if}\;i=1,3,\\ g_{i}^{R},\;\mathrm{if}\;i=2,4. \end{array}\right.$$

where $g_{i}$ can be calculated by Equation (10), and $g_{i}^{L}$ and $g_{i}^{R}$ are shown in Figure 3. We can get numerical flux $F_{c,i+1/2}^{\left(II*\right)}$ contributed by the normal velocity:

(18) $$F_{c,i+1/2}^{\left(II*\right)}={\left[\begin{array}{ccc} \rho U_{n} & \rho U_{n}U_{n}+p & \left(\rho \left(\frac{1}{2}U_{n}U_{n}+e\right)+p\right)U_{n} \end{array}\right]}^{T}=\sum_{i}\xi_{i}\varphi_{a}g_{i}(-\xi_{i}\delta t,t-\delta t),$$

(19) $$F_{c,i+1/2}^{\left(II*\right)}=\sum_{i}\xi_{i}\varphi_{\alpha }g_{i}(-\xi_{i}\delta t,t-\delta t)=\sum_{i=1,3}\xi_{i}\varphi_{\alpha }g_{i}^{L}+\sum_{i=2,4}\xi_{i}\varphi_{\alpha }g_{i}^{R}.)$$

From the above equation, the interface values of conservative variables can be calculated by:

(20) $$W{\left(0,t\right)}_{i+1/2}^{*}={\left[\begin{array}{ccc} \rho & \rho U_{n} & \frac{1}{2}\rho U_{n}U_{n}+\rho e \end{array}\right]}^{T}=\sum_{i}\varphi_{\alpha }g_{i}(0,t)=\sum_{i}\varphi_{\alpha }g_{i}\left(-\xi_{i}\delta t,t-\delta t\right),$$

(21) $$W{\left(0,t\right)}_{i+1/2}^{*}=\sum_{i=1,3}\varphi_{\alpha }g_{i}^{L}+\sum_{i=2,4}\varphi_{\alpha }g_{i}^{R}.)$$

Through the above formula, we can get the flow variable, combined with Equation (10), $g_{i}(0,t)$ can be easily calculated. Therefore, the two components of the flux $F_{c,i+1/2}^{\left(I\right)}$ and $F_{c,i+1/2}^{\left(II\right)}$ can be solved. The $\tau_{0}$ in Equation (16) is a coefficient used to control numerical dissipation. Yang [27] proposed to use a switching function to control A, as shown below:

(22) $$\tau_{0}=\tanh\left(m\frac{\left|p_{L}-p_{R}\right|}{p_{L}+p_{R}}\right).$$

Here, $p_{L}$ and $p_{R}$ denote the interface pressures. The parameter m is an empirical constant, and its value is set to 100 in this study.

**Figure 4:**
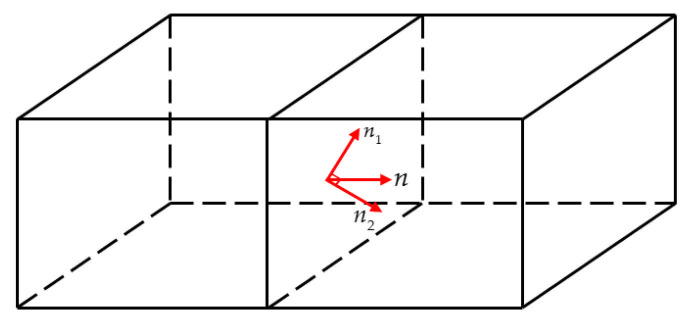
Schematic diagram of the decomposition of the normal vector at the 3D cell interface.

The interface normal vector in the RLBFS scheme is decomposed into two vectors $n_{1}$ and $n_{2}$, as shown in Figure 4. The final numerical flux is obtained by combining the fluxes in the two directions with appropriate weights [47]. The relationship between vectors $n_{1}$ and $n_{2}$ is as follows:

(23) $$\begin{array}{c} n_{1}\cdot n_{2}=0,\\ n=\alpha_{1}n_{1}+\alpha_{2}n_{2}, \end{array}$$

where $|n_{1}|=|n_{2}|=1$, $\alpha_{1}=n\cdot n_{1}$ and $\alpha_{2}=n\cdot n_{2}.$ The $\alpha_{1},\alpha_{2}\geq 0$ to ensure that $n_{1}$ and $n_{2}$ are always on one side of the interface. The numerical flux is calculated using the following weighted combination:

(24) $$F_{RLBFS}=F_{RLBFS}\left(n\right)=\alpha_{1}F_{LBFS}\left(n_{1}\right)+\alpha_{2}F_{LBFS}\left(n_{2}\right).$$

where $F_{LBFS}\left(n_{1}\right)$ and $F_{LBFS}\left(n_{2}\right)$ are determined using Equation (16). From the above formula, it is evident that $\alpha_{1}$ plays a crucial role, as its value directly influences the outcome of $F_{RLBFS}$. From $\alpha_{1}=n\cdot n_{1}$, we can know that $\alpha_{1}$ is directly determined by $n_{1}$, so, the value of $n_{1}$ is decisive for the result [48]. This paper adopts the results determined by Nishikawa et al. [49], as shown in the following formula:

(25) $$n_{1}=\left\{\begin{array}{ll} n, & \;\mathrm{if}\sqrt{{\left(\Delta u\right)}^{2}+{\left(\Delta v\right)}^{2}+{\left(\Delta w\right)}^{2}}\leq \varepsilon ,\\ \frac{\Delta ui+\Delta vj+\Delta wk}{\sqrt{{\left(\Delta u\right)}^{2}+{\left(\Delta v\right)}^{2}+{\left(\Delta w\right)}^{2}}}, & \;\mathrm{otherwise}.\; \end{array}\right.$$

where $\Delta (\;)={\left(\;\right)}_{R}-{\left(\;\right)}_{L}$, and when ε is close to 0, it will not affect the numerical result. On the contrary, it will have a more significant impact [48]. In this article, $\varepsilon =10^{-3}U^{*}$, $U^{*}$ is the free stream velocity. Equation (25) can determine the value of $n_{1}$, and the $n_{2}$ can be obtained by the following Equation:

(26) $$n_{2}=\left(n_{1}\times n\right)\times n_{1}.$$

#### 2.2.2. The URLBFS Developed Based on RLBFS

Following the above explanation of the RLBFS approach, it is evident that this method relies solely on the D1Q4 model for computing the normal numerical flux. RLBFS uses an approximate method to calculate the contribution of tangential velocity to momentum and energy fluxes, as shown in the following formula:

(27) $$\begin{array}{l} {\left(\rho U_{\tau }\right)}^{*}=\sum_{i}g_{i}\cdot U_{\tau }^{*}=\sum_{i=1,3}g_{i}^{L}\cdot U_{\tau }^{L}+\sum_{i=2,4}g_{i}^{R}\cdot U_{\tau }^{R},\\ {\left(\rho U_{n}U_{\tau }\right)}^{*}=\sum_{i}\xi_{i}g_{i}\cdot U_{\tau }^{*}=\sum_{i=1,3}\xi_{i}g_{i}^{L}\cdot U_{\tau }^{L}+\sum_{i=2,4}\xi_{i}g_{i}^{R}\cdot U_{\tau }^{R},\\ {\left(\rho U_{n}U_{\tau }^{2}\right)}^{*}=\sum_{i}\xi_{i}g_{i}\cdot {\left(U_{\tau }^{*}\right)}^{2}=\sum_{i=1,3}\xi_{i}g_{i}^{L}\cdot {\left(U_{\tau }^{L}\right)}^{2}+\sum_{i=2,4}\xi_{i}g_{i}^{R}\cdot {\left(U_{\tau }^{R}\right)}^{2}. \end{array}$$

$U_{\tau }^{*}$ is on the interface, and $U_{\tau }^{L}$ and $U_{\tau }^{R}$ are the left and right sides of the interface. Since this method relies on approximate calculations, it inevitably introduces some numerical dissipation, which hampers its ability to capture fine vortex structures and reduces the resolution of the flow field results. Especially when dealing with complex flow problems, numerical dissipation may lead to losing or distorting essential flow features. To achieve a numerical method with low dissipation, this study presents an enhanced scheme derived from the analytical solution of the Euler equations [35], which allows for the recalculation of a more accurate tangential velocity $U_{\tau }^{*}$. Specifically, this method first solves the interface normal velocity $U_{n}^{*}$ through Equation (28) and then uses the upwind direction of the $U_{n}^{*}$ to determine the value of the $U_{\Gamma }^{*}$.

(28) $$\begin{array}{l} \rho^{*}=\sum_{i=1,3}g_{i}^{L}+\sum_{i=2,4}g_{i}^{R},\\ \rho U_{n}^{*}=\sum_{i=1,3}\xi_{i}g_{i}^{L}+\sum_{i=2,4}\xi_{i}g_{i}^{R}. \end{array}$$

Let $U_{n}^{upwind}=U_{n}^{*}$, and get the new tangential velocity $U_{\tau }^{*}$ through the value of $U_{n}^{upwind}$, as shown below:

(29) $$U_{\tau }^{*}=\left\{\begin{array}{l} U_{\tau }^{L},\;\mathrm{if}\;U_{n}^{upwind}\geq 0,\\ U_{\tau }^{R},\;\mathrm{if}\;U_{n}^{upwind}<0. \end{array}\right.$$

So, Equation (27) can be reformulated as follows:

(30) $$\begin{array}{l} \left\{\begin{array}{l} {\left(\rho U_{\tau }\right)}^{*}=\sum_{i}g_{i}U_{\tau }^{*}=\sum_{i=1,3}g_{i}^{L}\cdot U_{\tau }^{L}+\sum_{i=2,4}g_{i}^{R}\cdot U_{\tau }^{L},\\ {\left(\rho U_{n}U_{\tau }\right)}^{*}=\sum_{i}\xi_{i}g_{i}U_{\tau }^{*}=\sum_{i=1,3}\xi_{i}g_{i}^{L}\cdot U_{\tau }^{L}+\sum_{i=2,4}\xi_{i}g_{i}^{R}\cdot U_{\tau }^{L},\\ {\left(\rho U_{n}U_{\tau }^{2}\right)}^{*}=\sum_{i}\xi_{i}g_{i}{\left(U_{\tau }^{*}\right)}^{2}=\sum_{i=1,3}\xi_{i}g_{i}^{L}\cdot {\left(U_{\tau }^{L}\right)}^{2}+\sum_{i=2,4}\xi_{i}g_{i}^{R}\cdot {\left(U_{\tau }^{L}\right)}^{2}, \end{array}\right.U_{n}^{upwind}\geq 0,\\ \left\{\begin{array}{l} {\left(\rho U_{\tau }\right)}^{*}=\sum_{i}g_{i}U_{\tau }^{*}=\sum_{i=1,3}g_{i}^{L}\cdot U_{\tau }^{R}+\sum_{i=2,4}g_{i}^{R}\cdot U_{\tau }^{R},\\ {\left(\rho U_{n}U_{\tau }\right)}^{*}=\sum_{i}\xi_{i}g_{i}U_{\tau }^{*}=\sum_{i=1,3}\xi_{i}g_{i}^{L}\cdot U_{\tau }^{R}+\sum_{i=2,4}\xi_{i}g_{i}^{R}\cdot U_{\tau }^{R},\\ {\left(\rho U_{n}U_{\tau }^{2}\right)}^{*}=\sum_{i}\xi_{i}g_{i}{\left(U_{\tau }^{*}\right)}^{2}=\sum_{i=1,3}\xi_{i}g_{i}^{L}\cdot {\left(U_{\tau }^{R}\right)}^{2}+\sum_{i=2,4}\xi_{i}g_{i}^{R}\cdot {\left(U_{\tau }^{R}\right)}^{2}, \end{array}\right.U_{n}^{upwind}<0. \end{array}$$

In this way, the URLBFS scheme provides more accurate tangential velocity, eliminates numerical errors introduced by approximate methods, reduce numerical dissipation, and enables the precise capture of both strong shock waves and small-scale vortices.

### 2.3. WENO Scheme Reconstruction Interface Flow Variables

Rather than applying WENO method directly to reconstruct interface fluxes as in standard FDM approaches, this work first uses WENO method to interpolate flow variables on each side of the interface and then feeds those values into the previously described URLBFS. As a result, the scheme achieves higher accuracy and lower numerical dissipation compared to conventional FDM.

#### 2.3.1. High-Order WENO Reconstruction Method

**Figure 5:**
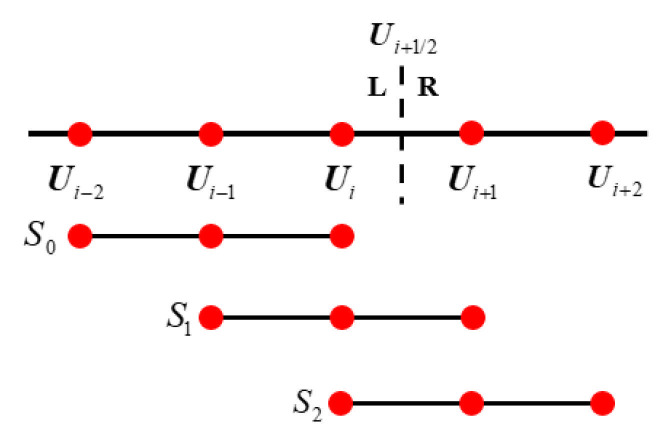
Global template and sub-templates of WENO5. Red dots represent the cell nodes.

This section focuses on WENO5 and WENO7 interpolation methods. In order to get the physical quantity on the interface’s left side, $\rho^{-},u^{-},v^{-},w^{-},p^{-}$, the WENO5 scheme needs to use the physical information on the five nodes around the interface, $S=\left\{U_{i-2},U_{i-1},U_{i},U_{i+1},U_{i+2}\right\}$, as shown in Figure 5.

It is divided into three sub-templates to construct low-order reconstruction polynomials:

(31) $$S_{0}=\left\{U_{i-2},U_{i-1},U_{i}\right\},\;S_{1}=\left\{U_{i-1},U_{i},U_{i+1}\right\},\;S_{2}=\left\{U_{i},U_{i+1},U_{i+2}\right\}.$$

After weighted combination of these three formulas, the final WENO5 reconstruction solution is obtained:

(32) $$\begin{array}{ll} U_{i+1/2}^{L} & =w_{0}\left(\frac{1}{3}U_{i-2}-\frac{7}{6}U_{i-1}+\frac{11}{6}U_{i}\right)\\ & +w_{1}\left(-\frac{1}{6}U_{i-1}+\frac{5}{6}U_{i}+\frac{1}{3}U_{i+1}\right)\\ & +w_{2}\left(\frac{1}{3}U_{i}+\frac{5}{6}U_{i+1}-\frac{1}{6}U_{i+2}\right), \end{array}$$

The weight coefficient and smoothness index proposed by Shu Jianjun [8] are used as follows:

(33) $$w_{k}=\frac{\alpha_{k}}{\alpha_{0}+\alpha_{1}+\alpha_{2}},\;\alpha_{k}=\frac{d_{k}}{{\left(\beta_{k}+\epsilon \right)}^{2}},\;k=0,1,2.$$

(34) $$\begin{array}{c} \beta_{0}=\frac{13}{12}{\left(U_{i-2}-2U_{i-1}+U_{i}\right)}^{2}+\frac{1}{4}{\left(U_{i-2}-4U_{i-1}+3U_{i}\right)}^{2},\\ \beta_{1}=\frac{13}{12}{\left(U_{i-1}-2U_{i}+U_{i+1}\right)}^{2}+\frac{1}{4}{\left(U_{i-1}-U_{i+1}\right)}^{2},\\ \beta_{2}=\frac{13}{12}{\left(U_{i}-2U_{i+1}+U_{i+2}\right)}^{2}+\frac{1}{4}{\left(3U_{i}-4U_{i+1}+U_{i+2}\right)}^{2}. \end{array}$$

The optimal linear weighting coefficient is given by [50]:

(35) $$d_{0}=\frac{1}{10},d_{1}=\frac{3}{5},d_{2}=\frac{3}{10},$$

Set $\varepsilon =10^{-6}$ to ensure that the denominator is non-zero. Only the process of calculating $U_{i+1/2}^{L}$ is given above. The value $U_{i+1/2}^{R}$ on the right side can be obtained symmetrically and thus will not be discussed in detail here.

**Figure 6:**
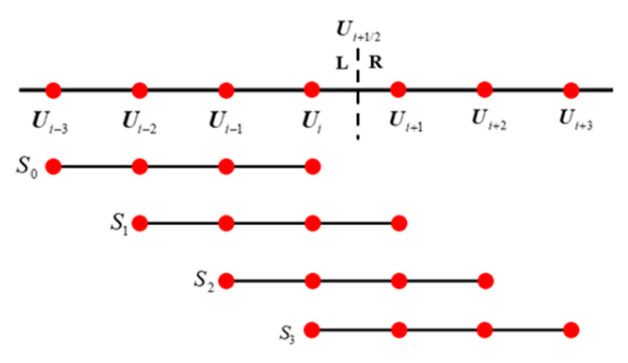
Global template and sub-templates of WENO7. Red dots represent the cell nodes.

The global template in the WENO7 scheme contains seven surrounding points, $S=\left\{U_{i-3},\cdots ,U_{i+3}\right\}$, and is divided into four sub-stencils in total, as shown in the Figure 6 below.

The left side flow variables of the interface are reconstructed using WENO7 as follows:

(36) $$\begin{array}{cc} U_{i+1/2}^{L} & =w_{0}\left(-\frac{1}{4}U_{i-3}+\frac{13}{12}U_{i-2}-\frac{23}{12}U_{i-1}+\frac{25}{12}U_{i}\right)\\ & +w_{1}\left(\frac{1}{12}U_{i-2}-\frac{5}{12}U_{i-1}+\frac{13}{12}U_{i}+\frac{1}{4}U_{i+1}\right)\\ & +w_{2}\left(-\frac{1}{12}U_{i-1}+\frac{7}{12}U_{i}+\frac{7}{12}U_{i+1}-\frac{1}{12}U_{i+2}\right)\\ & +w_{3}\left(\frac{1}{4}U_{i}+\frac{13}{12}U_{i+1}-\frac{5}{12}U_{i+2}+\frac{1}{12}U_{i+3}\right), \end{array}$$

The Detailed parameters are shown below:

(37) $$w_{k}=\frac{\alpha_{k}}{\alpha_{0}+\alpha_{1}+\alpha_{2}+\alpha_{3}},\;\alpha_{k}=\frac{d_{k}}{{\left(\beta_{k}+\epsilon \right)}^{2}},\;k=0,1,2,3.$$

(38) $$\begin{array}{ll} \beta_{0} & =U_{i-3}(547U_{i-3}-3882U_{i-2}+4642U_{i-1}-1854U_{i})\;\\ & \;\;+U_{i-2}(7043U_{i-2}-17,246U_{i-1}+7042U_{i})\;\\ & \;\;+U_{i-1}(11,003U_{i-1}-9402U_{i})+U_{i}(2107U_{i}),\\ \beta_{1} & =U_{i-2}(267U_{i-2}-1642U_{i-1}+1602U_{i}-494U_{i+1})\;\\ & \;\;+U_{i-1}(2843U_{i-1}-5966U_{i}+1922U_{i+1})\;\\ & \;\;+U_{i}(3443U_{i}-2522U_{i+1})+U_{i+1}(547U_{i+1}),\\ \beta_{2} & =U_{i-1}(547U_{i-1}-2522U_{i}+1922U_{i+1}-494U_{i+2})\\ & \;\;+U_{i}(3443U_{i}-5966U_{i+1}+1602U_{i+2})\\ & \;\;+U_{i+1}(2843U_{i+1}-1642U_{i+2})+U_{i+2}(267U_{i+2}),\\ \beta_{3} & =U_{i}(2107U_{i}-9402U_{i+1}+7042U_{i+2}-1854U_{i+3})\\ & \;\;+U_{i+1}(11,003U_{i+1}-17,246U_{i+2}+4642U_{i+3})\\ & \;\;+U_{i+2}(7043U_{i+2}-3882U_{i+3})+U_{i+3}(547U_{i+3}). \end{array}$$

(39) $$d_{0}=\frac{1}{35},d_{1}=\frac{12}{35},d_{2}=\frac{18}{35},d_{3}=\frac{4}{35}.$$

The ε has the same values as WENO5, and only the interpolation results of the left variables are shown. The variables on the right only need symmetric operations.

#### 2.3.2. Characteristic Space

In compressible flow problems, oscillations often occur at shocks and strong discontinuities, which may cause divergent results [51]. To better solve these complex flow problems, we put the reconstruction process in the feature space. For convenience of description, we only show the Jacobian matrix in the x direction:

(40) $$A(U)=\frac{\partial F}{\partial U}{|}_{i+1/2}=\left[\begin{array}{ccccc} 0 & 1 & 0 & 0 & 0\\ \hat{\gamma }H-u^{2}-c^{2} & (3-\gamma )u & -\hat{\gamma }v & -\hat{\gamma }w & \hat{\gamma }\\ -uv & v & u & 0 & 0\\ -uw & w & 0 & u & 0\\ \frac{1}{2}u[(\gamma -3)H-c^{2}] & H-\hat{\gamma }u^{2} & -\hat{\gamma }uv & -\hat{\gamma }uw & \gamma u \end{array}\right].$$

where $\hat{\gamma }=\gamma -1$, the speed of sound $c=\sqrt{\gamma p/\rho }\varepsilon$. The total enthalpy Hε as shown below:

(41) $$H=\frac{1}{2}V^{2}+\frac{c^{2}}{\gamma -1},$$

where $V=\left(u^{2}+v^{2}+w^{2}\right)$. The right eigenvectors of the A(U) is:

(42) $$\begin{array}{l} \;R^{F}=\left(\begin{array}{ccccc} 1 & 1 & 0 & 0 & 1\\ u-c & u & 0 & 0 & u+c\\ v & v & 0 & -c & v\\ w & w & c & 0 & w\\ H-uc & 1/2V^{2} & cw & -cv & H+uc \end{array}\right), \end{array}$$

The matrix of left eigenvectors, $L^{F}A(U)$, is simply ${\left(R^{F}\right)}^{-1}$. The conservative variables $U_{i+1/2,j,k}$ at $x_{i+1/2,j,k}$ is:

(43) $$U_{i+1/2,j,k}=\frac{1}{2}\left(U_{i,j,k}+U_{i+1,j,k}\right),$$

The conservation variable in physical space can be transformed into characteristic space by multiplying the eigenvector $L^{F}$ on the left, as shown below:

(44) $$V_{m,j,k}=L^{F}U_{m,j,k},$$

where k represents number of nodes required for interpolation reconstruction. After the eigenspace is reconstructed by the WENO scheme to obtain the value $V_{i+1/2,j,k}^{\pm }$ at the interface, then go to the physical space through the right eigenvector $R^{F}$ as follows:

(45) $$U_{i+1/2,j,k}^{\pm }=R^{F}V_{i+1/2,j,k}^{\pm }.$$

The conservation variables are obtained through the above process, and flow variables can be easily derived. The methods for the other two directions are the same and will not be repeated here.

## 3. GPU Implementations

In previous research on LBFS and RLBFS, the calculations were mainly based on CPU implementation, and some of the work accelerated the calculations by introducing OpenMP instructions and a message passing interface (MPI). However, the acceleration effect is insignificant due to the limitation of the number of CPU threads. Unlike traditional CPUs, the GPU contains many arithmetic logic units (ALUs) or threads. The number of threads is much higher than that of the CPU; so, they are suitable for parallel computing large-scale simple programs. OpenCL and CUDA are the two main programming models for processing GPU parallel tasks. This study utilizes the CUDA platform for GPU programming to fully leverage the highly parallel computing capabilities of GPU, thereby significantly improving computational efficiency.

### 3.1. CUDA-GPU Introduction

**Figure 7:**
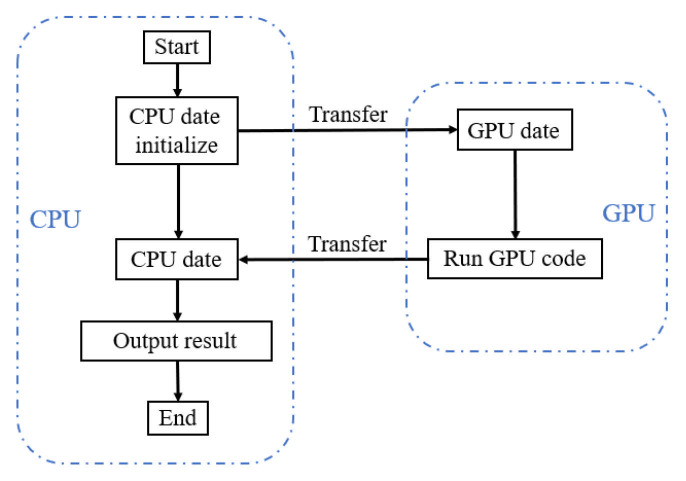
A heterogeneous program model that uses CPU and GPU together.

**Figure 8:**
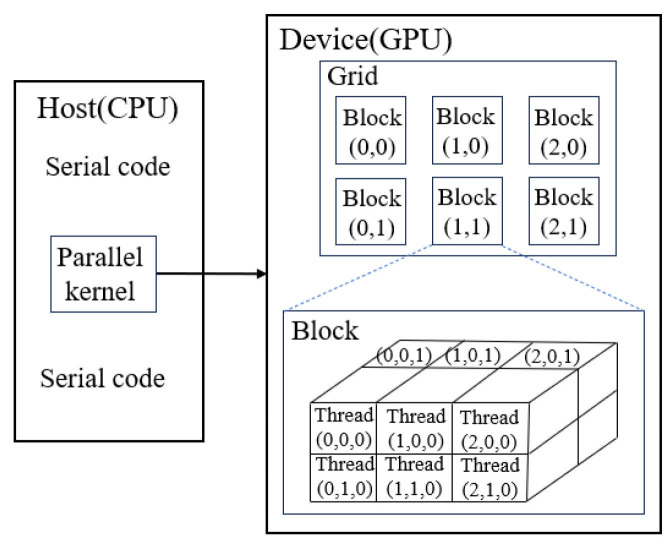
Description of thread hierarchy.

The original program was written in Fortran 90 to solve the compressible flow. In order to use GPU to accelerate the calculation, the Fortran program needs to be rewritten as a version that supports CUDA. There are usually two options: CUDA Fortran (PGI) and CUDA C (NVIDIA). In this study, to fully utilize the functions of CUDA to achieve efficient GPU computing, we chose to rewrite the Fortran program using CUDA C. In a heterogeneous computing environment, the CPU and GPU each undertake different tasks to achieve parallel computing better. Generally, the CPU is suitable for processing complex logical calculations and program control, while the GPU is good at large-scale simple computing parallel tasks. Therefore, it is necessary to adopt a heterogeneous programming model. In CUDA programming, the code is divided into serial and parallel parts. The code running on the host (CPU) prepares the necessary data, such as variable declaration, initialization, and data output operations. The host side then transfers the data to the device (GPU), responsible for performing computationally intensive tasks. Once the GPU completes the task, the result is returned to the host side through data transfer for subsequent processing. The overall process is shown in Figure 7. The code that runs on the GPU is commonly referred to as a “kernel.” These parallel codes are executed simultaneously by hundreds or even thousands of threads on the device. These threads are divided and grouped into multiple thread blocks, which can be further organized into different thread block grids based on the characteristics of the hardware, as shown in Figure 8. To simplify programming, each thread is generally assigned a unique thread index, which can be organized into a 1D, 2D, or 3D thread block structure. The threads and thread block number should be reasonably selected based on hardware performance and computing requirements to maximize the use of GPU resources. Too few threads or thread blocks will lead to underutilization of hardware resources, while too many threads or thread blocks may cause resource competition and performance degradation. Therefore, choosing the appropriate thread and thread block configuration is the key to optimizing GPU computing performance.

### 3.2. Parallel Implementation of the WENO-URLBFS Scheme

**Figure 9:**
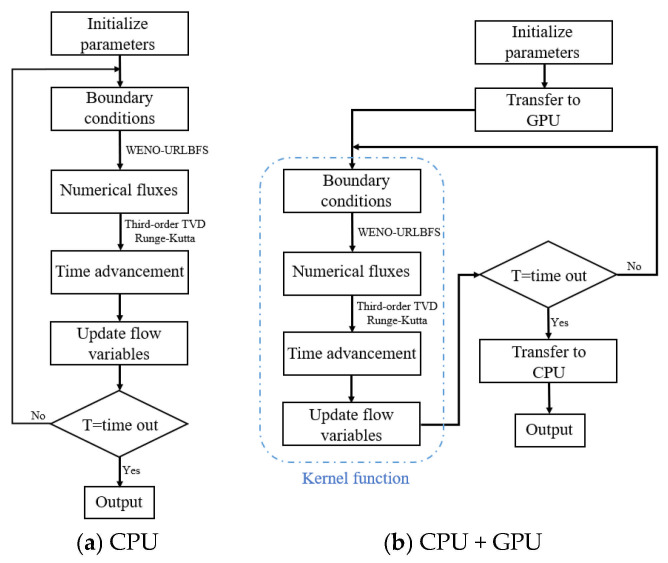
Comparison between the CPU-only serial implementation and the CPU + GPU parallel execution of the WENO-URLBFS scheme.

This paper uses NVIDIA TITAN V GPU and CUDA C programming model to develop WENO-URLBFS GPU parallel code. The parameters of the TITAN V graphics card are shown in Table 1. Solving the compressible flow problem using the FD-WENO-URLBFS scheme consists of three key parts: (1) flow field initialization; (2) boundary conditions, solution flux, and time advancement; (3) checking the simulation time and outputting the results, as shown in Figure 9a. The first part assigns values to the flow field through initial conditions and defines some global parameters. This part is only executed once at the beginning of the program and has little impact on the calculation time; so, it does not need to be parallelized. The second part focuses on solving the entire flow field, evaluating the numerical fluxes using the URLBFS scheme, thus ensuring that the flow characteristics are accurately captured. After obtaining the spatial discrete operator, the TVD Runge-Kutta method performs time advancement and obtains the flow variables of the next time step. Since this part requires millions of calculations on each grid interface, the amount of calculation is vast and occupies a large part of the whole calculation time; so, this part needs to be parallelized. The last part is determining whether the calculation has reached the termination time. If it has, the data of the entire flow field is output. Since this part is only executed once at the end of the calculation, it does not need to be parallelized.

The second part basically occupies the entire computing time; so, it is feasible to parallelize it. The above description shows that the GPU is just a computing device, and its work requires commands from the host. The host implements the device’s operation by calling the kernel function. The kernel function of the second part is implemented in parallel on the GPU as a kernel function, and its specific process is shown in Figure 9b. An important feature of a kernel function is that it allows for the allocation of multiple threads. Therefore, the number of threads must be reasonably specified before calling the kernel function. For three-dimensional flow problems, the thread configuration can be defined by $grid\_size\left(Gx,Gy,Gz\right)$ and $block\_size\left(Bx,By,Bz\right)$. Among them, grid_size represents grid size, and block_size represents thread block size, and the product of these two variables is total number of threads. Higher computational efficiency in GPUs is achieved only when the number of threads exceeds the available computing cores, allowing for more effective utilization of the available computational resources. Each thread has a unique identity in the kernel function that determines the computing cell for which the thread is responsible. Taking the three-dimensional problem as an example, the index corresponding to the computational cell in the x-direction can be calculated as follows:

(46) nx=blockDim.x×blockIdx.x+threadIdx.x

where blockDim.x represents the threads number in the thread block, corresponding to the value of block_size.Bx; blockIdx.x is the index of the thread block within the grid; and blockIdx.x denotes the thread’s position inside its block. Each thread can uniquely locate a specific unit in the calculation grid through the above indexing mechanism. After implementing the above content, parallel computing can be realized, and both CPU and GPU codes use double precision; so, there is no precision mismatch problem.

## 4. Numerical Tests and Validation

This section verifies the numerical accuracy of the WENO-URLBFS scheme and evaluates the performance of its GPU implementation through the density perturbation advection problem. Additionally, test cases such as Inviscid Taylor–Green Vortex, Explosion in a Box, Explosion in an Enclosed Cabin and Oblique shock–mixing layer interaction are used to further illustrate the URLBFS scheme’s ability to capture detailed flow structures and its low-dissipation properties. Unless otherwise specified, all the following test cases use the 3rd TVD Runge-Kutta for time advancement with a constant time step of 1 × 10^−4^. All the test cases in this study were conducted on a Windows desktop equipped with a Hygon 7185 CPU (2.0 GHz) and an NVIDIA TITAN V GPU. The specifications of the CPU and GPU are listed in Table 1. The GPU-accelerated code was developed using CUDA C++, while the CPU-based version was implemented in standard C++. Both versions were compiled in double-precision mode. The CPU-based flow simulations presented in this paper were executed in single-thread mode, which is a common performance analysis method reported in literature [52,53,54].

### 4.1. Advection of Density Perturbation Problem

This example tests the WENO-URLBFS numerical scheme precision and compares the efficiency of CPU serial and GPU parallel approaches. The initial conditions are that all parameters except density are set to 1, where density is [55]:

(47) $$\rho \left(x,y,z\right)=1+0.2\sin\left(\pi \left(x+y+z\right)\right),$$

The exact solution is:

(48) $$\rho \left(x,y,z,t\right)=1+0.2\sin\left(\pi \left(x+y+z-t\right)\right),$$

**Figure 10:**
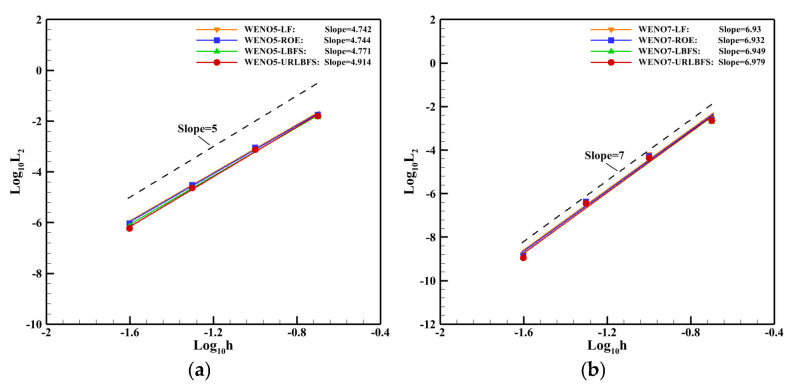
L_2_ error between the density and the grid size h. (**a**): WENO5 reconstruction method; (**b**): WENO7 reconstruction method.

This example employs a [0, 2]^3^ computational domain with periodic boundary conditions applied to all boundaries. The simulation runs for a total time of t = 2. The table below presents the density error and the order of accuracy for different flux solvers, utilizing different WENO reconstruction methods at grid sizes of 1/5, 1/10, 1/20, and 1/40. Figure 10 illustrates the relationship between the L_2_ error and grid resolution for various numerical schemes. The accuracy of these schemes can be fitted using the least squares method. It is evident that the different flux calculation methods, founded on the finite difference framework proposed in this paper, generally achieve the expected fifth and seventh-order accuracies, with the URLBFS scheme exhibiting slightly higher accuracy than the other three schemes. Table 2 and Table 3 presents the density errors and the corresponding segmented orders of accuracy for various numerical schemes across different grid resolutions. It can be seen that URLBFS has a smaller error, indicating that this scheme has lower numerical dissipation.

**Figure 11:**
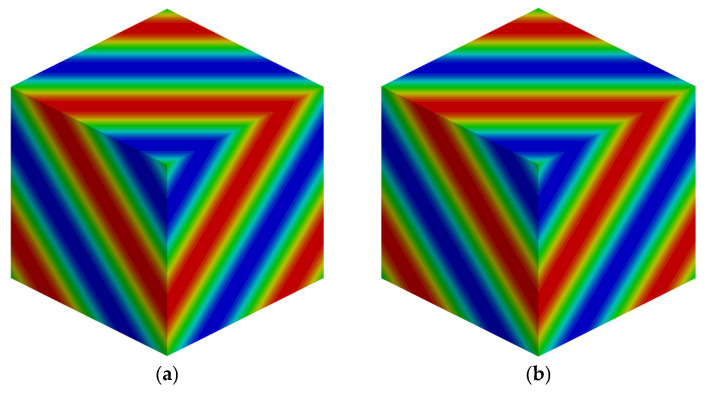
Comparison of CPU and GPU results for the density perturbation advection problem with 403 grid points using the WENO5-URLBFS scheme. (**a**): CPU serial; (**b**): GPU parallel.

**Figure 12:**
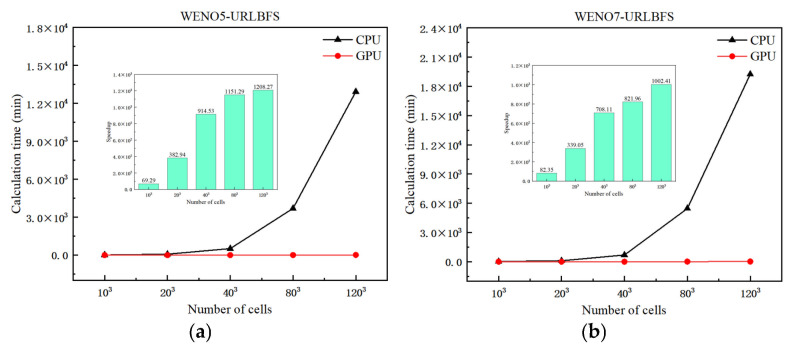
Speedup of GPU parallel scheme relative to CPU serial scheme at different meshes. (**a**): WENO5-URLBFS scheme; (**b**): WENO7-URLBFS scheme.

**Figure 13:**
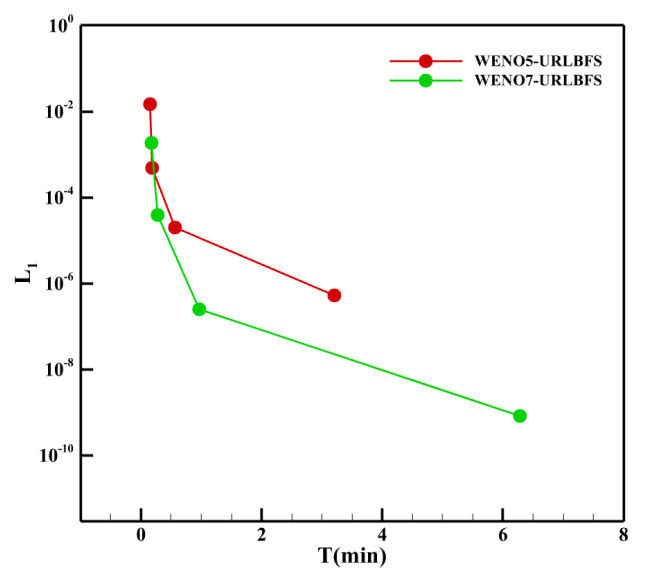
Comparison of accuracy and computational cost of different reconstruction schemes at various grid resolutions.

To quantitatively evaluate the computational performance of the FD-WENO-URLBFS scheme on both CPU and GPU platforms, we use a fixed time step for time advancement, and the $dt=1\times 10^{-4}$ is used. The CPU code is calculated using the Hygon x86 7185-32c CPU and C++ compiler, while the GPU calculation is completed using the Nvidia TITAN V graphics card, combined with Nvidia CUDA technology and NVCC compiler, both using double precision calculations. Figure 11 presents the density contour distributions obtained from the serial and parallel implementations at a grid resolution of 40^3^. Table 4 further verifies the consistency between the CPU and GPU results by comparing the L_1_ and L_2_ errors. The results demonstrate that the parallel implementation produces highly consistent and reliable solutions comparable to those of the serial version. The next will focus on analyzing the acceleration efficiency of the parallel scheme for various grid sizes. The speedup is introduced to represent the GPU acceleration efficiency, as shown below

(49) $$Speedup=\frac{T_{CPU}}{T_{GPU}}$$

where $T_{CPU}$ and $T_{GPU}$ denote the computation time required by the CPU and GPU code, respectively. Table 5 lists the computation times for CPU and GPU implementations, as well as the corresponding speedup ratios, for the WENO-URLBFS scheme across different grid sizes. Figure 12 provides a visual comparison of computation time across different grid sizes, along with the GPU’s speedup relative to the CPU. From the table and figures, it is evident that as the grid number increases exponentially, the computation time for CPU serial solving grows significantly, posing a substantial challenge for research work that requires obtaining results within a limited time. In contrast, the GPU parallel solution shows excellent acceleration performance. Under the same WENO reconstruction method, as the grid amount increases, the acceleration effect of the GPU becomes more significant, especially in the WENO5 method, where the GPU achieves a speedup ratio of more than 1208.27 times. It is essential to highlight that, for the same grid size, the increase in numerical accuracy has a more noticeable impact on CPU computation time, while the GPU can still complete the calculation efficiently, reflecting its advantages in processing complex and high-precision calculations. Figure 13 presents a comparison of computational time and numerical accuracy between the WENO5 and WENO7 reconstruction schemes under the GPU implementation at various grid resolutions, aiming to evaluate which scheme achieves higher computational efficiency on the GPU. The circular markers in the figure represent different grid sizes, namely 103, 203, 403, and 803. As shown in the figure, when the grid resolution is relatively low, the WENO7 reconstruction scheme exhibits higher overall efficiency, considering both computational time and numerical accuracy. As the grid resolution increases, the WENO7 scheme continues to achieve smaller numerical errors; however, its advantage in computational time over WENO5 becomes less pronounced compared with that observed at lower resolutions.

### 4.2. Inviscid Taylor–Green Vortex

The velocity and pressure distribution at the initial moment of this example presents a symmetrical vortex structure. As time goes by, the inviscid vortex begins to stretch and gradually produces smaller and smaller scales. This problem is commonly utilized to prove the scheme’s potential to catch high-frequency vortices while examining properties like the conservation of total kinetic energy and numerical dissipation. The simulations are performed in the region [0, 2π]^3^ with a grid resolution of 128^3^. The initial conditions are [56]:

(50) $$\left\{\begin{array}{l} \rho =1,\\ u\left(x,y,z\right)=V_{0}\sin\left(x/L\right)\cos\left(y/L\right)\cos\left(z/L\right),\\ v\left(x,y,z\right)=-V_{0}\cos\left(x/L\right)\sin\left(y/L\right)\cos\left(z/L\right),\\ w\left(x,y,z\right)=0,\\ p\left(x,y,z\right)=p_{0}+\frac{\rho_{0}V_{0}^{2}}{16}\left(\left[\cos\left(2x/L\right)+\cos\left(2y/L\right)\right]\left[\cos\left(2z/L\right)+2\right]-2\right). \end{array}\right.$$

**Figure 14:**
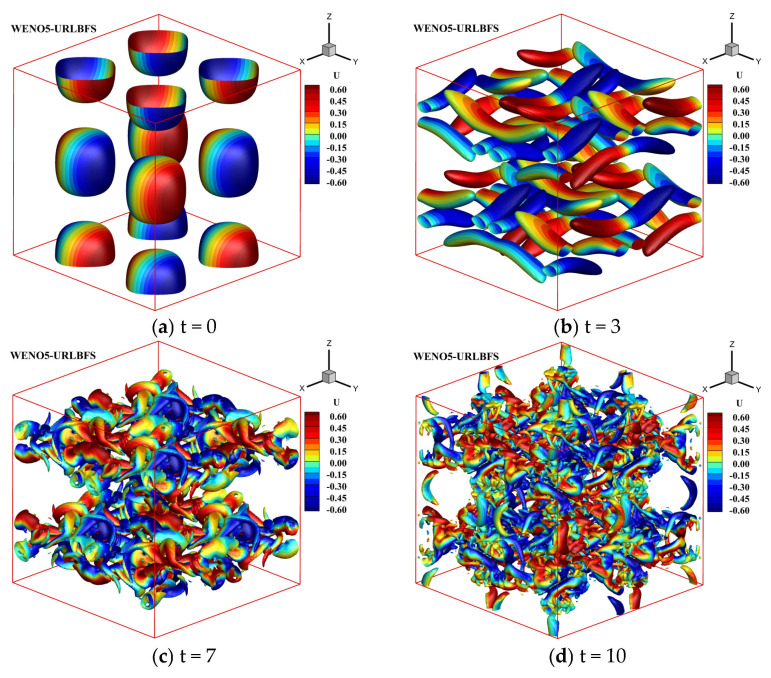
Temporal evolution of an inviscid Taylor–Green vortex based on the WENO5-URLBFS scheme, using Q-criterion iso-surfaces to show the vortex structure and colored according to the velocity in the x direction.

All boundaries are subjected to periodic boundary conditions. Since the total time is T = 10, to save time, the actual CPU calculation time to t = 0.1 is counted and proportionally converted to the total calculation time of t = 10 to estimate the total CPU calculation time. Table 6 shows the computational time of the WENO-URLBFS scheme for simulating the Inviscid Taylor–Green Vortex problem under CPU serial computing and GPU parallel computing conditions, where the GPU achieves a speedup ratio of up to 1063.49. Figure 14 presents the time-dependent evolution of the vortex. When t = 0, the vorticity diagram shows a set of symmetrical vortex structures forming a regular grid arrangement. As time goes by, the vortex structure becomes more complex, and the vortex diagram shows that the vortex gradually strengthens or splits. New vortex pairs or the interaction between the vortex and other flow field features appears. When t = 10 is reached, the vorticity diagram shows the asymmetry of the vortex structure, the originally regular vortex pattern is disturbed or distorted, and the size of the vortex also changes in space, which is manifested as different vortex intensities at different positions.

**Figure 15:**
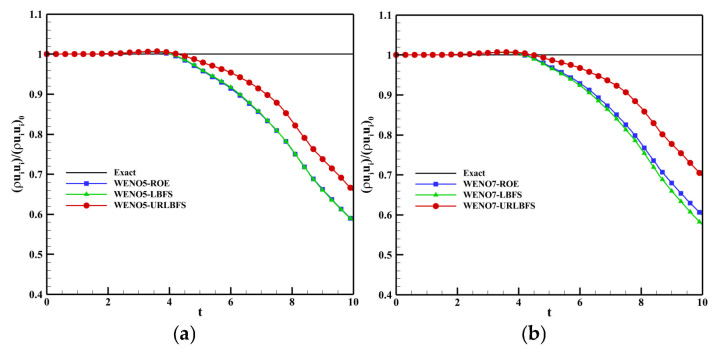
3D inviscid Taylor-Green vortex problem. Time history of the ratio between the average kinetic energy and its initial value for various numerical schemes. Panel (**a**) shows the WENO5 reconstruction technique; Panel (**b**) shows the WENO7 reconstruction technique.

**Figure 16:**
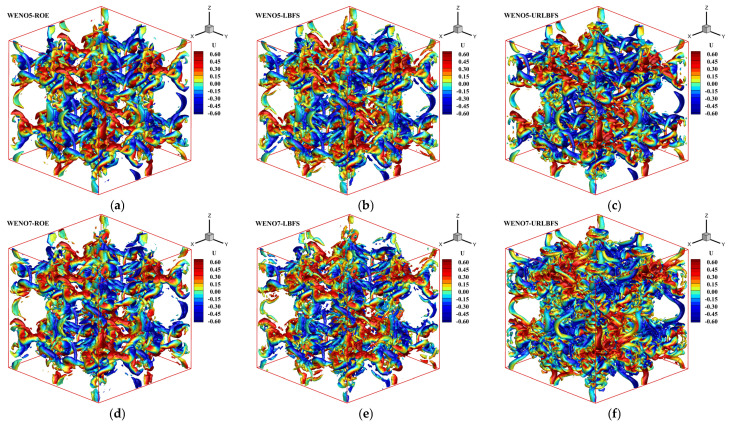
3D inviscid Taylor-Green vortex problem. Iso-surfaces of Q-criterion at Q = 2 for different numerical schemes colored with x-velocity at t = 10. Panel (**a**–**c**): WENO5 reconstruction technique; Panel (**d**–**f**): WENO7 reconstruction technique.

Figure 15 shows the variation in the ratio of kinetic energy relative to its initial value ($(\rho u_{i}u_{i})/{\left(\rho u_{i}u_{i}\right)}_{0}$) with time when using various numerical schemes. The results indicate that the proposed URLBFS scheme outperforms the ROE and LBFS schemes in kinetic energy recovery, demonstrating its low numerical dissipation characteristics. Figure 16 shows the Q criterion iso-surfaces at Q = 2 for different solvers. The results indicate that the URLBFS scheme resolves finer vortex structures, providing higher flow field resolution.

### 4.3. Explosion in a Box

**Figure 17:**
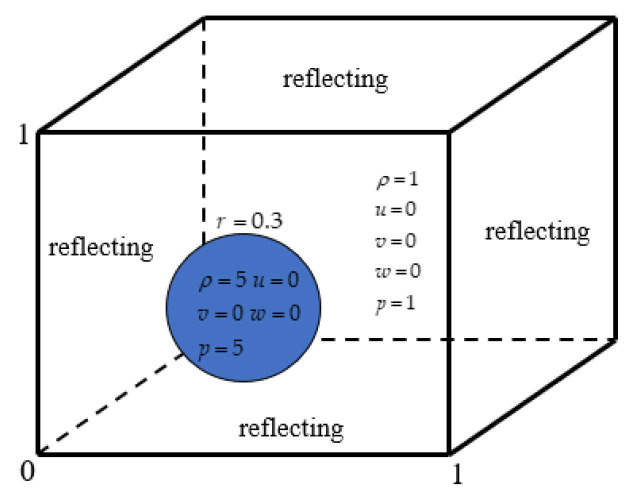
Schematic diagram of the initial conditions. The blue circle represents an initially stationary, high-density, high-pressure bubble surrounded by ambient gas.

This problem describes the diffusion of a spherical shock wave generated through an explosion in a closed box, and its interaction with the wall of the box produces a series of complex flow phenomena [57]. The schematic diagram is shown in Figure 17 below, a sphere with a radius of 0.3 is in the square box and the calculation domain size is [0, 1]^3^, which is discretized into a uniform grid of 120^3^. All boundaries are set as reflection conditions, and the center of the sphere is (0.4,0.4,0.4). Initial conditions are given by:

(51) $$(\rho ,u,v,w,p)=\left\{\begin{array}{ll} \left(5,0,0,0,5\right) & if\;r\leq 0.3,\\ \left(1,0,0,0,1\right) & else. \end{array}\right.$$

**Figure 18:**
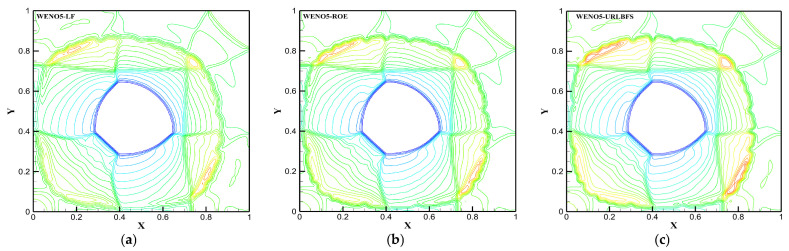

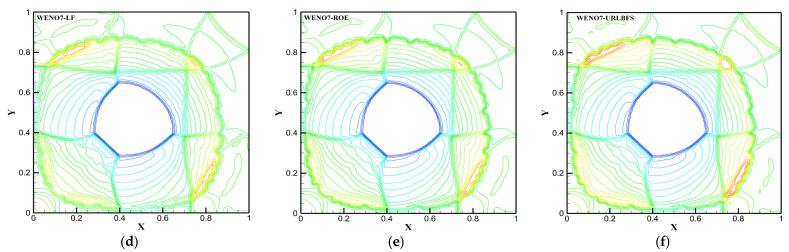
Density distributions on z = 0.4 were visualized as 23 equally spaced contour levels from 0.2 to 2.4 using different schemes. Panel (**a**–**c**): WENO5 reconstruction technique; Panel (**d**–**f**): WENO7 reconstruction technique.

**Figure 19:**
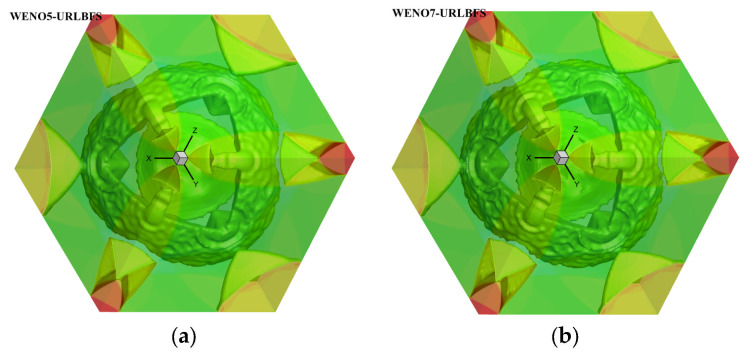
Density iso-surface (ρ=1.8) contours were obtained using the WENO-URLBFS schemes. Panel (**a**) shows the WENO5 reconstruction technique; Panel (**b**) shows the WENO7 reconstruction technique.

**Figure 20:**
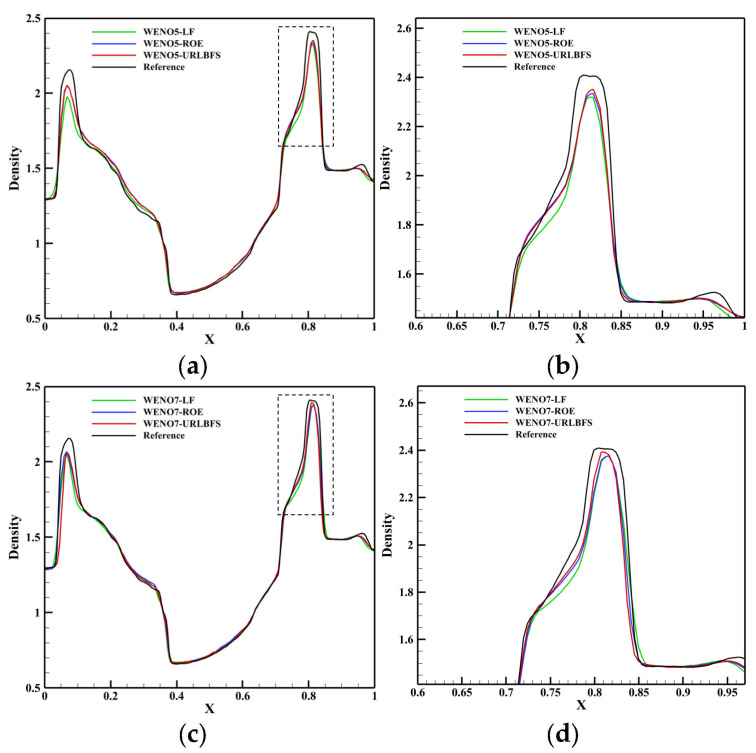
Comparison of density profiles along y = 0.2 on the plane z = 0.4 obtained using different numerical schemes. Panel (**a**,**c**) represent the reconstruction technologies of WENO5 and WENO7, respectively; Panel (**b**,**d**) are locally enlarged views.

Table 7 compares the calculation time of the GPU parallel implementation of the WENO-URLBFS scheme and the CPU serial solution in the Explosion in a Box problem. The results indicate that the GPU parallel solution has a prominent advantage in computational efficiency, and its speedup ratio can reach 1289.62. It effectively demonstrates the efficiency of GPU parallel methods in solving complex flow problems. Figure 18 is the density contour at t = 0.5 on z = 0.4 plane, illustrating the density distribution under different schemes. Figure 19 shows the density (ρ=1.8) of iso-surfaces under present schemes. The figure shows that the FD-WENO framework proposed in this paper performs well under different solvers and can solve complex flow phenomena stably. To provide a more intuitive comparison of the results obtained by different numerical schemes, Figure 20 presents the density distribution along y = 0.2 on the plane z = 0.4. The reference solution was obtained by using the WENO5–ROE scheme with an eight-fold increase in the number of grid cells. As shown in the figure, the URLBFS scheme exhibits larger amplitude variations in the density profile, indicating its significantly lower numerical dissipation. The enhanced amplitude preservation further demonstrates the superior capability of the URLBFS scheme in resolving flow features and capturing fine-scale structures.

### 4.4. Explosion in an Enclosed Cabin

**Figure 21:**
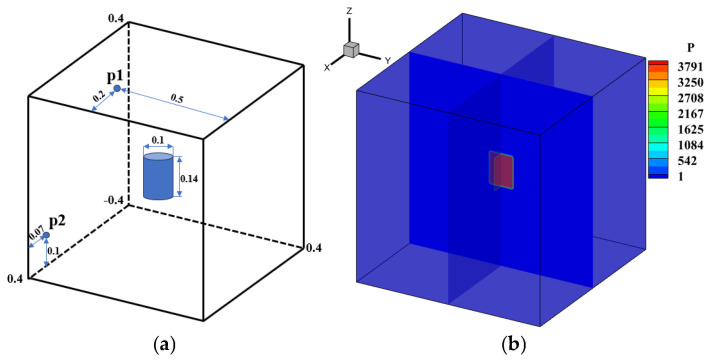
Schematic showing the confined chamber setup and the initial explosion region. Panel (**a**): Geometric configuration and the computational setup. The Blue dots represent monitoring points. The interior of the blue cylinder represents a high-density, high-pressure environment. Panel (**b**): Pressure distribution contour map at the initial moment.

In order to further extend the applicability of URLBFS, this study employs the method to perform numerical simulations of the propagation of explosive waves generated by a cylindrical high-pressure, high-density gas within a confined square chamber, and deeply explores the evolution law of the wave system and the pressure load characteristics of typical measuring points on the wall. By meticulously constructing the geometric model and boundary conditions of the enclosed chamber, this study analyzes the propagation, reflection, and interaction mechanisms of the explosive wave in the restricted space, thereby revealing its dynamic evolution under multiple reflections and interferences. To simulate the explosion process, high-pressure and high-density gas is set in the blue cylindrical area, and the surrounding area represents the air domain. As shown in Figure 21. The specific initial conditions and schematic diagram are as follows [58]:

(52) $$(\rho ,u,v,w,p)=\left\{\begin{array}{c} \left(166.3,0,0,0,3791\right)\\ \left(1,0,0,0,1\right) \end{array}\begin{array}{c} if\sqrt{{\left(x-0.4\right)}^{2}+{\left(y-0.4\right)}^{2}}\leq 0.05\;\left|z\right|\leq 0.07,\;\\ else. \end{array}\right.$$

Points p1 and p2 are two pressure measurement points set on the wall surface, which are used to compare the differences in calculations using different numerical schemes. The simulations are performed in the region [−0.4, 0.4]^3^ with a grid resolution of 100^3^. All boundaries are set as reflection boundary conditions.

**Figure 22:**
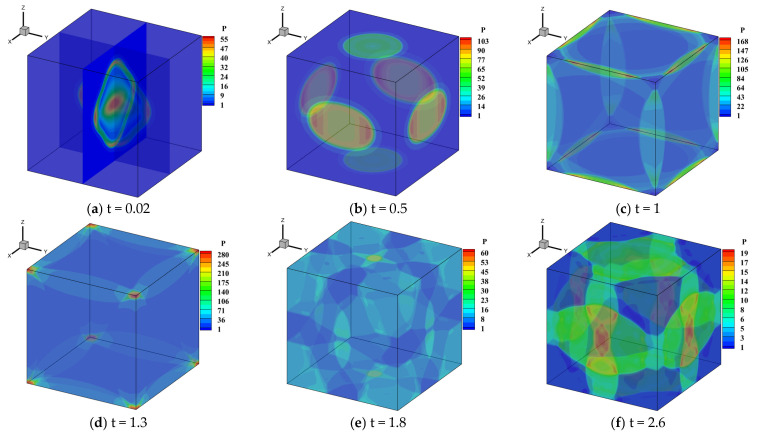
Temporal evolution of the pressure contour maps for an explosion in a confined chamber simulated using the WENO5-URLBFS scheme.

Table 8 presents the execution times of the URLBFS scheme for CPU serial and GPU parallel implementations. The GPU implementation speedup ratio is up to 1198.11, which fully demonstrates the significant advantages of GPU in computationally intensive numerical simulations. The pressure contour of the initial explosion in the three-dimensional closed cabin simulated by the WENO5-URLBFS scheme is shown in Figure 22. The figure provides into how the explosion wave propagates during the initial phase of the blast. The high-pressure gas first expands freely in a three-dimensional cylindrical symmetric manner. When the explosion wave first touches the cabin wall, a regular reflection occurs (see Figure 22a,b). Due to the constraints of the surrounding walls, the explosion wave has a local pressure concentration phenomenon at the intersection of the two walls (see Figure 22c). When the explosion wave propagates further to the corner of the cabin, a more significant pressure convergence phenomenon is formed in the area where the three walls meet (see Figure 22d). Subsequently, the shock waves produced by the reflection of the cabin wall propagate toward the center of the cabin and converge and collide with each other in this area (see Figure 22e,f). Analysis reveals that the explosion wave within the closed square cabin undergoes repeated wall reflections, center convergence, collisions, and subsequent re-reflections. During this process, the intensity of the explosion wave gradually decays, and the pressure in the cabin tends to be uniform.

**Figure 23:**
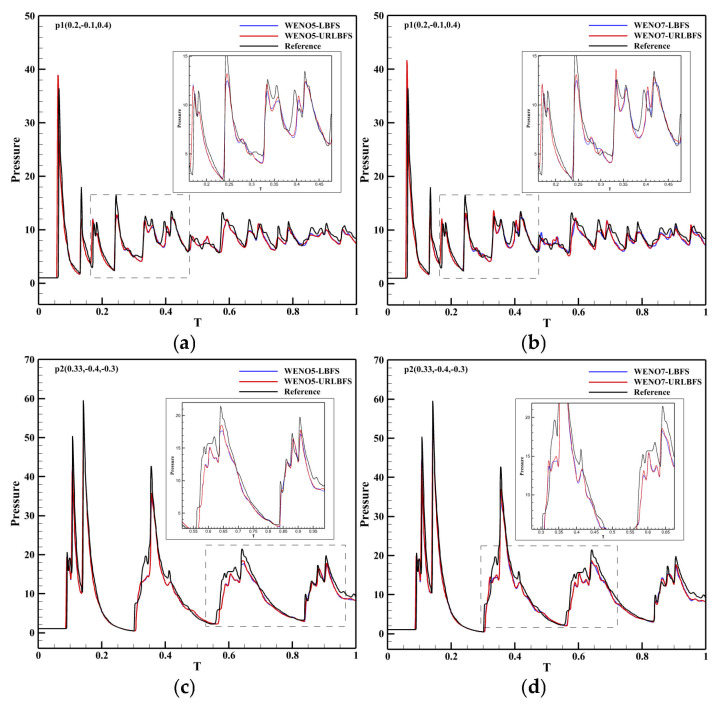
Comparison curves of the pressure change in p1 and p2 measurement points with simulation time in different schemes. Panel (**a**,**c**) represent the reconstruction technologies of WENO5 and WENO7, respectively; Panel (**b**,**d**) are locally enlarged views.

Figure 23 compares the pressure time histories at two monitoring points at t = 1. Owing to the combined effects of wall reflections and multiple interactions of the blast-induced shock waves, the pressure response inside the three-dimensional enclosed chamber exhibits a multi-peak pattern with gradual attenuation over time. The reference solution was obtained by using the WENO5–LBFS scheme with an eight-fold increase in the number of grid cells. As illustrated in the figure, the URLBFS scheme yields pressure profiles with larger amplitude, indicating its lower numerical dissipation and its superior ability to capture detailed wave fluctuations and local extrema. Overall, URLBFS demonstrates enhanced resolution of nonlinear wave interactions in this complex three-dimensional blast scenario.

### 4.5. Oblique Shock–Mixing Layer Interaction

This example verifies the robustness of the scheme in complex flow conditions where the distinction between shock waves and smooth flow is unclear. This example shows a shock wave approaching from the upper left corner, angled 12° relative to the flow direction. The shock wave interacts with the shear layer, destabilizing it and leading to the formation of a vortex structure. These vortices gradually rotate along the flow direction as time changes, developing a complex shock wave structure. The initial condition as follows [59]:

(53) $$\left(\rho ,u,v,w,p\right)=\left\{\begin{array}{l} \left(0.3626,2,0,0,0.3327\right),y\leq 0\\ \left(1.6374,3,0,0,0.3327\right),y>0 \end{array}\right.$$

The simulations are performed in the region [0, 200] × [−20, 20] × [−20, 20] with a grid resolution of 400 × 80 × 80. The inflow velocity applied to the left boundary is:

(54) $$\begin{array}{l} u_{in}=2.5+0.5\tanh\left(2y\right)\\ v_{in}=\sum_{k=1}^{2}a_{k}^{\prime }\cos\left(\frac{2\pi kt}{T}+\frac{z}{L_{z}}+\phi_{k}\right)e^{-\frac{y^{2}}{10}} \end{array}$$

where $a_{1}=a_{2}=0.05$, $\phi_{1}=0,\phi_{2}=\pi /2$. $T=\lambda /u_{c}$, the λ=30 and the $u_{c}=2.68$. Zero gradient extrapolation is used at the right boundary. A slip wall condition is applied at the bottom boundary, while the upper boundary adopts a post-shock value of $\left(\rho ,u,v,p\right)=\left(2.1101,2.9709,-0.1367,0.4754\right)$. The front and rear walls along the Z direction are set as symmetric boundary conditions. Here, Pr = 0.72 and Re = 500. Since a fixed time step is used, the CPU serial and GPU parallel computing times are compared at t = 1 as shown in Table 9. The results show that the GPU parallel speedup ratio is up to 1002.82.

**Figure 24:**
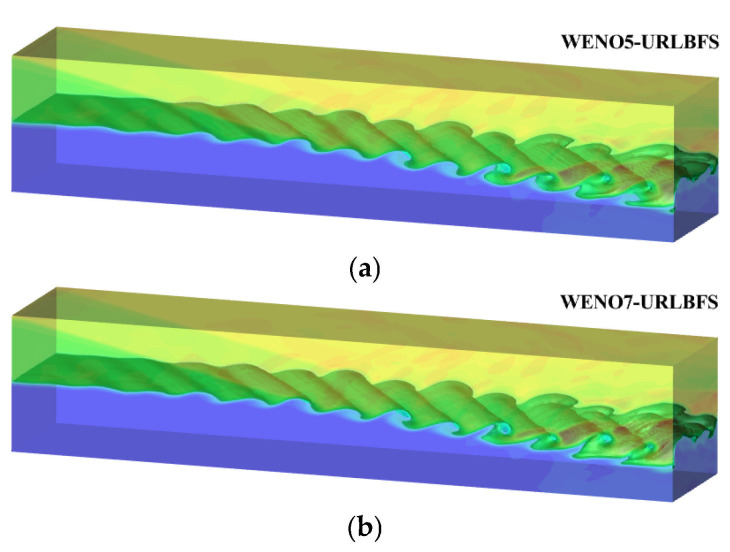
Three-Dimensional density iso-surface plot for the oblique shock mixing layer interaction problem. Panel (**a**) shows the WENO5 reconstruction technique; Panel (**b**) shows the WENO7 reconstruction technique.

Apply velocity disturbance on the left boundary to establish velocity inlet boundary condition. This velocity disturbance will produce a phase difference in the *z* direction, resulting in a clearly regular vortex structure before the incident shock wave interacts with the shear layer. After the shock wave collides with the shear layer, the spanwise vortex undergoes significant deformation. Due to the lower boundary being a slip wall, the shock wave reflects upon impact, leading to additional interactions that further evolve and distort the vortex structure. Figure 24 illustrates the density contour at t = 120 calculated using the present scheme.

**Figure 25:**
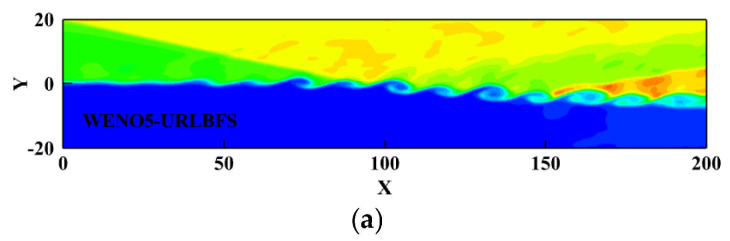

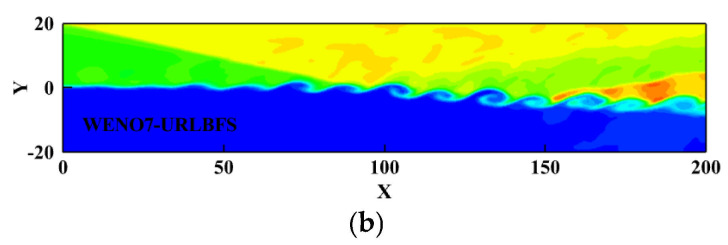
Density contours with 23 equally spaced contour lines ranging from 0.4 to 2.4 on plane z = 20 were obtained using the WENO-URLBFS schemes. Panel (**a**) shows the WENO5 reconstruction technique; Panel (**b**) shows the WENO7 reconstruction technique.

**Figure 26:**
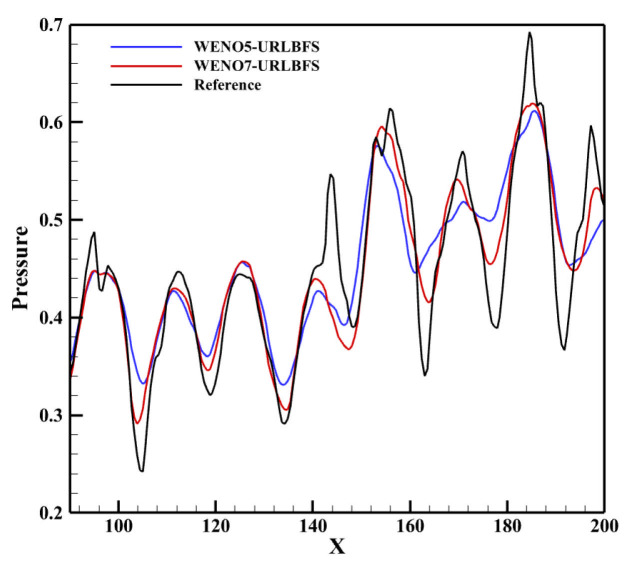
Computed pressure distributions along a straight line across the vortices from point (90, 0, 20) to point (200, −6, 20).

Figure 25 presents the density contours at the z = 20 cross-section obtained using the WENO-URLBFS scheme. The interaction between the spanwise vortex structure and the shock wave is clearly captured, and the evolution of the vortex generated by the interference between the oblique shock and the boundary layer is accurately reproduced. To compare the results of the two schemes intuitively, the pressure distributions along a straight line across the vortices from point (90, 0, 20) to point (200, −6, 20) are shown and compared with the reference solution in Figure 26. The reference solution was obtained by using the WENO5–URLBFS scheme with an eight-fold increase in the number of grid cells. The comparison of the pressure profiles shows that the solution based on the WENO7 reconstruction exhibits larger amplitude and is closer to the reference solution, indicating lower numerical dissipation. This observation is consistent with the accuracy assessments presented earlier.

## 5. Conclusions

This paper presents WENO-URLBFS, a high-order, low-dissipation numerical scheme with GPU-parallel acceleration, for simulating 3D compressible flows involving strong shock waves and contact discontinuities. The scheme improves upon the conventional LBFS and RLBFS approaches by replacing the approximate treatment of the interface tangential velocity with the exact Euler equation solution, thereby effectively reducing numerical dissipation. Unlike traditional finite difference methods that directly reconstruct interface fluxes, WENO-URLBFS reconstructs interface physical quantities using the WENO scheme in characteristic space and then evaluates the numerical fluxes via the URLBFS solver. The implementation on the CUDA platform enables efficient GPU parallelization, resulting in a substantial enhancement in computational performance.

The accuracy of the WENO-URLBFS scheme is initially assessed by simulating the advection of a density perturbation, achieving formal fifth- and seventh-order convergence. In this test case, the consistency between CPU serial and GPU parallel implementations is confirmed, and the GPU acceleration efficiency is assessed across different grid resolutions, yielding a maximum speedup ratio of 1208.27. The low-dissipation property of the scheme is quantitatively demonstrated in the inviscid Taylor vortex problem by analyzing the temporal evolution of the total kinetic energy. Furthermore, several challenging three-dimensional problems—including the explosion in a box, explosion in an enclosed cabin, and the oblique shock mixing layer interaction—are simulated to validate both the computational efficiency and accuracy of the scheme in capturing complex flow phenomena. For these cases, GPU acceleration performance is also compared. Although variations in initial conditions and grid sizes lead to differences in acceleration efficiency, the speedup generally reaches around 1000. Overall, the results confirm that the GPU-parallel WENO-URLBFS scheme can efficiently and robustly capture essential physical features such as strong shock waves, vortices, and discontinuities while maintaining low numerical dissipation, thereby demonstrating its strong capability for high-dimensional, strongly nonlinear compressible flow problems.

Currently, the numerical experiments are mainly performed on a single GPU device. Future work will aim to further enhance the accuracy of the WENO reconstruction, explore multi-GPU strategies for greater speedup, and develop high-order multi-GPU schemes suitable for non-uniform grid problems.

## Figures and Tables

**Table 1:** Specifications of the Hygon 7185 CPU and NVIDIA TITAN V GPU.

		Hygon 7185	NVIDIA TITAN V
Processor	Total number of cores	32	5120
	Clock rate	2.0 GHz	1455 MHz
Memory	Global memory	64 GB	12 GB
	Shared memory	--	64 KB
	Registers per block	--	256 KB
Peak theoretical performance	Floating point operations	1-core: 32 GFLOP/s	7450 GFLOP/s
	Memory bandwidth	170 GB/s	652.8 GB/s

**Table 2:** Comparison of error and numerical order for WENO5-based flux solvers at different grid resolutions.

Schemes	h	L_1_ Error	L_1_ Order	L_2_ Error	L_2_ Order	L_∞_ Error	L_∞_ Order
WENO5-LF	1/5	1.76 × 10^−2^	-	1.89 × 10^−2^	-	2.53 × 10^−2^	-
	1/10	9.19 × 10^−4^	4.256	9.88 × 10^−4^	4.259	1.40 × 10^−3^	4.179
	1/20	2.94 × 10^−5^	4.968	3.33 × 10^−5^	4.891	5.15 × 10^−5^	4.761
	1/40	9.08 × 10^−7^	5.015	1.02 × 10^−6^	5.026	1.67 × 10^−6^	4.943
WENO5-ROE	1/5	1.63 × 10^−2^	-	1.75 × 10^−2^	-	2.33 × 10^−2^	-
	1/10	8.45 × 10^−4^	4.266	9.12 × 10^−4^	4.262	1.30 × 10^−3^	4.161
	1/20	2.68 × 10^−5^	4.979	3.05 × 10^−5^	4.902	4.81 × 10^−5^	4.760
	1/40	8.35 × 10^−7^	5.004	9.43 × 10^−7^	5.016	1.57 × 10^−6^	4.935
WENO5-LBFS	1/5	1.36 × 10^−2^	-	1.47 × 10^−2^	-	2.06 × 10^−2^	-
	1/10	6.91 × 10^−4^	4.298	7.55 × 10^−4^	4.281	1.11 × 10^−3^	4.211
	1/20	2.13 × 10^−5^	5.018	2.45 × 10^−5^	4.946	4.06 × 10^−5^	4.779
	1/40	6.64 × 10^−7^	5.005	7.51 × 10^−7^	5.028	1.31 × 10^−6^	4.956
WENO5-URLBFS	1/5	1.47 × 10^−2^	-	1.58 × 10^−2^	-	2.20 × 10^−2^	-
	1/10	6.78 × 10^−4^	4.436	7.39 × 10^−4^	4.419	1.09 × 10^−3^	4.337
	1/20	2.00 × 10^−5^	5.083	2.28 × 10^−5^	5.021	3.60 × 10^−5^	4.919
	1/40	5.26 × 10^−7^	5.248	5.92 × 10^−7^	5.267	9.04 × 10^−7^	5.317

**Table 3:** Comparison of error and numerical order for WENO7-based flux solvers at different grid resolutions.

Schemes	h	L_1_ Error	L_1_ Order	L_2_ Error	L_2_ Order	L_∞_ Error	L_∞_ Order
WENO7-LF	1/5	1.76 × 10^−2^	-	1.89 × 10^−2^	-	2.53 × 10^−2^	-
	1/10	9.19 × 10^−4^	4.256	9.88 × 10^−4^	4.259	1.40 × 10^−3^	4.179
	1/20	2.94 × 10^−5^	4.968	3.33 × 10^−5^	4.891	5.15 × 10^−5^	4.761
	1/40	9.08 × 10^−7^	5.015	1.02 × 10^−6^	5.026	1.67 × 10^−6^	4.943
WENO7-ROE	1/5	2.12 × 10^−3^	-	2.52 × 10^−3^	-	3.98 × 10^−3^	-
	1/10	4.68 × 10^−5^	5.502	5.32 × 10^−5^	5.564	9.84 × 10^−5^	5.336
	1/20	3.06 × 10^−7^	7.257	4.28 × 10^−7^	6.958	1.07 × 10^−6^	6.517
	1/40	1.02 × 10^−9^	8.235	1.39 × 10^−9^	8.263	3.48 × 10^−9^	8.270
WENO7-LBFS	1/5	1.73 × 10^−3^	-	2.08 × 10^−3^	-	3.38 × 10^−3^	-
	1/10	3.95 × 10^−5^	5.451	4.52 × 10^−5^	5.526	8.43 × 10^−5^	5.327
	1/20	2.53 × 10^−7^	7.286	3.52 × 10^−7^	7.006	9.07 × 10^−7^	6.538
	1/40	8.09 × 10^−10^	8.289	1.12 × 10^−9^	8.297	2.93× 10^−9^	8.276
WENO7-URLBFS	1/5	1.85 × 10^−3^	-	2.23 × 10^−3^	-	3.69 × 10^−3^	-
	1/10	3.95 × 10^−5^	5.451	4.52 × 10^−5^	5.526	8.43 × 10^−5^	5.327
	1/20	2.53 × 10^−7^	7.286	3.52 × 10^−7^	7.006	9.07 × 10^−7^	6.538
	1/40	8.09 × 10^−10^	8.289	1.12 × 10^−9^	8.297	2.93 × 10^−9^	8.276

**Table 4:** Consistency comparison of the WENO-URLBFS scheme results between CPU and GPU implementations.

Schemes	h	CPU (L_1_)	GPU (L_1_)	CPU (L_2_)	GPU (L_2_)
WENO5-URLBFS	1/20	2.00 × 10^−5^	2.01 × 10^−5^	2.28 × 10^−5^	2.29 × 10^−5^
	1/40	5.26 × 10^−7^	5.28 × 10^−7^	5.92 × 10^−7^	5.94 × 10^−7^
WENO7-URLBFS	1/20	2.53 × 10^−7^	2.53 × 10^−7^	3.52 × 10^−7^	3.53 × 10^−7^
	1/40	8.09 × 10^−10^	8.10 × 10^−10^	1.12 × 10^−9^	1.12 × 10^−9^

**Table 5:** Computation time and speedup ratio of CPU serial and GPU parallel in WENO-URLBFS scheme at different meshes.

Scheme	Mesh Resolution	CPU (min)	GPU (min)	Speedup
WENO5-URLBFS	1/5	10.24	0.15	69.29
	1/10	68.93	0.18	382.94
	1/20	512.14	0.56	914.53
	1/40	3695.64	3.21	1151.29
	1/60	12,928.54	10.7	1208.27
WENO7-URLBFS	1/5	14.00	0.17	82.35
	1/10	94.93	0.28	339.05
	1/20	686.87	0.97	708.11
	1/40	5461.92	6.28	821.96
	1/60	19,246.23	19.2	1002.41

**Table 6:** Comparison of CPU serial and GPU parallel computation times for the WENO-URLBFS scheme at t = 10.

Scheme	Number of Cells	CPU (min)	GPU (min)	Speedup
WENO5-URLBFS	128^3^	77,630.52	91.42	849.16
WENO7-URLBFS		135,786.3	127.68	1063.49

**Table 7:** Comparison of CPU serial and GPU parallel computation times for the WENO-URLBFS scheme at t = 0.5.

Scheme	Number of Cells	CPU (min)	GPU (min)	Speedup
WENO5-URLBFS	120^3^	2914.55	2.26	1289.62
WENO7-URLBFS		4534.52	4.46	1016.70

**Table 8:** Comparison of CPU serial and GPU parallel computation times for the WENO-URLBFS scheme at t = 2.6.

Scheme	Number of Cells	CPU (min)	GPU (min)	Speedup
WENO5-URLBFS	100^3^	1569.52	1.31	1198.11
WENO7-URLBFS		2342.25	2.32	1009.59

**Table 9:** Comparison of CPU serial and GPU parallel computation times for the WENO-URLBFS scheme at t = 1.0.

Scheme	Number of Cells	CPU (min)	GPU (min)	Speedup
WENO5-URLBFS	400 × 80 × 80	12,214.31	12.18	1002.82
WENO7-URLBFS		15,582.72	15.91	979.43

## Data Availability

The original contributions presented in this study are included in this article; further inquiries can be directed to the corresponding author.
